# Interpretable Machine Learning to Predict Metformin-Induced Vitamin B12 Deficiency: Association with Glycemic Control and Neuropathic Symptoms

**DOI:** 10.3390/metabo16040227

**Published:** 2026-03-30

**Authors:** Yasmine Salhi, Meriem Yazidi, Amine Dhraief, Elyes Kamoun, Melika Chihaoui, Tamim Alsuliman, Layth Sliman

**Affiliations:** 1 ISITCom, University of Sousse, Sousse 4011, Tunisia; yassminsalhi999@gmail.com; 2 La Rabta Hospital, Department of Endocrinology, Faculty of Medicine of Tunis, University of Tunis el Manar, Tunis 1007, Tunisia; elyes.kamoun@fmt.utm.tn (E.K.); melika.chihaoui@fmt.utm.tn (M.C.); 3 ESEN, University of Manouba, La Manouba 2010, Tunisia; amine.dhraief@esen.tn; 4 Oncology-Hematology Department, Saint-Quentin Hospital, 02100 Saint-Quentin, France; dr.tameem.soliman@gmail.com; 5 Efrei Research Lab, Paris Panthéon Assas University, 94800 Villejuif, France; layth.sliman@gmail.com

**Keywords:** type 2 diabetes, metformin, vitamin B12 deficiency, artificial intelligence, XGBoost, SHAP

## Abstract

**Background/Objectives:** Vitamin B12 deficiency is a common but often underdiagnosed complication in patients with type 2 diabetes (T2D) undergoing long-term metformin therapy. Accurate early prediction could enable targeted screening and timely intervention. This study aimed to develop and interpret a machine learning model for predicting vitamin B12 deficiency in metformin-treated patients with T2D, using eXtreme Gradient Boosting (XGBoost). **Methods:** A retrospective cross-sectional study was conducted at a single endocrinology centre (La Rabta University Hospital, Tunis, Tunisia). Patients with T2D treated with metformin for at least three years were included (*n* = 257); those with conditions independently affecting vitamin B12 metabolism were excluded. Vitamin B12 deficiency was defined as a serum B12 level below 150 pmol/L or a borderline level (150–221 pmol/L) with concurrent hyperhomocysteinemia (>15 μmol/L). XGBoost was selected after comparison with Logistic Regression (L2), Random Forest, and Support Vector Machine on the same 5-fold stratified cross-validated pipeline. Hyperparameters were optimized via Bayesian search (100 iterations × 5-fold stratified cross-validation), with the Matthews correlation coefficient (*MCC*) as the primary optimization metric to account for class imbalance. Model interpretability was achieved using SHapley Additive exPlanations (SHAP). Discrimination and calibration were assessed on an independent test set using bootstrap 95% confidence intervals (2000 resamples). **Results:** Of 257 patients, 95 (37.0%) presented with vitamin B12 deficiency. On the independent test set (*n* = 52), the optimized XGBoost model achieved an ROC-AUC of 0.671 [95% CI: 0.514–0.818], sensitivity of 0.737 [95% CI: 0.533–0.938], specificity of 0.545 [95% CI: 0.375–0.710], *MCC* of 0.273 [95% CI: 0.018–0.517], and a Brier Score of 0.259. SHAP analysis identified HbA1c, microalbuminuria, autonomic neuropathy, BMI, DN4 score, and fasting glucose as the most influential predictors. Nonlinear SHAP interaction plots revealed an increased predicted risk in patients with low HbA1c combined with a high cumulative metformin dose. **Conclusions:** The XGBoost–SHAP framework provided interpretable predictions of vitamin B12 deficiency in patients with T2D on metformin, identifying key clinical profiles for targeted screening. External multi-centre validation is required before clinical deployment.

## 1. Introduction

The prevalence of type 2 diabetes (T2D) is continuously increasing worldwide. Metformin is widely used in the treatment of T2D due to its efficacy, low cost, safety profile, and low risk of hypoglycemia. However, in recent years, metformin has been linked to an increased risk of vitamin B12 deficiency [1,2]. This condition, which may lead to severe complications, is often underestimated. Moreover, tests to detect it are not systematically performed, as they are considered costly or reserved for cases with obvious symptoms. Since 2017, the American Diabetes Association (ADA) has recommended regular vitamin B12 screening in individuals treated with metformin [3]. Despite this, real-world data indicate that screening practices remain suboptimal, as shown in recent observational studies [4]. Furthermore, most scientific societies have not issued explicit recommendations on which patient subgroups should be prioritized for screening [5,6,7]. Current approaches—often based solely on treatment duration and metformin doses—are insufficient to capture the complex interplay of clinical and biochemical factors contributing to deficiency risk. There is therefore a need for more precise, data-driven strategies that can integrate a wide range of patient information to improve prediction accuracy and guide preventive interventions. Such an approach would help optimize resource allocation, target screening efforts, and reduce delays in management. Machine learning approaches have emerged as promising tools in this context. Algorithms such as eXtreme Gradient Boosting (XGBoost) [8] can model nonlinear associations and interactions among numerous variables, making them well-suited for clinical prediction tasks. Moreover, interpretability techniques like SHapley Additive exPlanations (SHAP) can provide transparency by quantifying the influence of each predictor on individual predictions, supporting clinical decision-making. This study aimed to develop and interpret an XGBoost-based predictive model to identify vitamin B12 deficiency in patients with T2D receiving metformin therapy, using a comprehensive set of demographic, clinical, and laboratory variables.

## 2. Materials and Methods

### 2.1. Study Population

The study population was drawn from a previously published investigation that aimed to assess the prevalence of vitamin B12 deficiency in a Tunisian population with T2D on metformin treatment and to identify associated risk factors [9]. It was a cross-sectional study carried out in the Endocrinology department of the Rabta University Hospital in Tunis (Tunisia) between September 2019 and June 2020. Sociodemographic, clinical, biological, and therapeutic data were collected from 257 patients with T2D treated with metformin for at least 3 years. A minimum duration of 3 years of metformin therapy was set as an inclusion criterion, given the latency in the development of vitamin B12 deficiency attributable to hepatic stores. Patients with conditions that could affect vitamin B12 metabolism, such as alcoholism, Biermer’s anemia, malabsorption syndromes, neoplasia, severe renal impairment (eGFR < 30 mL/min/1.73 m^2^), thyroid disorders, pregnancy, a vegetarian diet, or those who receive vitamin B12 supplementation, were excluded from the study. Furthermore, patients with vitamin B12 deficiency were excluded if they tested positive for antiparietal cell or anti-intrinsic factor antibodies.

### 2.2. Data Collection

The collected data included sociodemographic parameters, diabetes history, and associated diseases. Adherence to metformin therapy was evaluated using the Girerd questionnaire [10]. The cumulative dose of metformin was calculated by multiplying the daily dose of metformin (in grams) by the duration of metformin use (in days). The “Metformin Usage Index” (MUI), defined by Shivaprasad et al. [11] to quantify metformin exposure, was calculated by the formula: (daily metformin dose in milligrams × duration of metformin intake in years)/1000. Other antidiabetic treatments potentially used in combination with metformin, as well as the long-term use of acid-lowering agents, were documented. Anthropometric parameters (weight, height) were measured, and body mass index (BMI) was calculated. Blood pressure (BP) was taken in supine and standing positions. Peripheral neuropathy was diagnosed based on a Neuropathic Pain 4 questions (DN4) score ≥ 4/10 [12], distal hypoesthesia on the 10 g monofilament test, and/or absent osteotendinous reflexes in the lower limbs. Autonomic neuropathy was considered in the presence of erectile dysfunction in men, gastroparesis (early satiety or postprandial fullness with nausea/vomiting), motor diarrhea (≥3 loose stools/day for ≥4 weeks without identifiable cause), resting tachycardia, or documented orthostatic hypotension (drop in systolic BP ≥ 20 mmHg or diastolic BP ≥ 10 mmHg within 3 min of standing).

A 12 h fasting blood sample was collected to measure vitamin B12, homocysteine, anti-parietal cell, anti-intrinsic factor antibodies, fasting blood glucose, glycated hemoglobin (HbA1c), complete blood count (CBC), and estimated glomerular filtration rate (eGFR), calculated using the MDRD (Modification of Diet in Renal Disease) formula [13]. Vitamin B12 and homocysteine were measured using an immunoassay method on the ARCHITECT analyzer (Abbott Laboratories, Abbott Park, IL, USA). Anti-parietal cell and anti-intrinsic factor antibodies were analyzed using the ELISA (Enzyme-Linked Immunosorbent Assay) technique (Euroimmun AG, Lübeck, Germany). CBC was performed with the Sysmex XN-1000 analyzer (Sysmex Corporation, Kobe, Japan) and HbA1c was measured using an enzymatic method. The 24 h microalbuminuria value and the fundus examination results from the past year were collected from the patient’s medical records. The study received ethical approval from La Rabta University Hospital Ethics Committee, and informed consent was obtained from each participant prior to their involvement.

### 2.3. Definition of Vitamin B12 Deficiency

Vitamin B12 deficiency was defined as a serum vitamin B12 level below 150 pmol/L (203 pg/mL), or as a borderline level (150–221 pmol/L) with concurrent hyperhomocysteinemia (homocysteine > 15 μmol/L). This definition was guided by expert recommendations [5,14] and findings from our previous study, which demonstrated a significant inverse correlation between vitamin B12 and homocysteine levels (r = −0.2; *p* = 0.001) in this population [9]. Homocysteine is a sensitive marker of vitamin B12 deficiency, with early elevations occurring as vitamin B12 levels decline, potentially preceding both the appearance of clinical symptoms and a marked reduction in vitamin B12 concentrations [5,14]. An elevated homocysteine level in the context of a borderline-normal serum vitamin B12 concentration may reflect a functional intracellular vitamin B12 deficiency that is not detected by serum vitamin B12 measurement alone [14,15]. Incorporating hyperhomocysteinemia into the definition of vitamin B12 deficiency for individuals exhibiting borderline vitamin B12 levels helps avoid the misdiagnosis of functional and intracellular deficiencies.

### 2.4. Data Preprocessing

**Figure 1:**
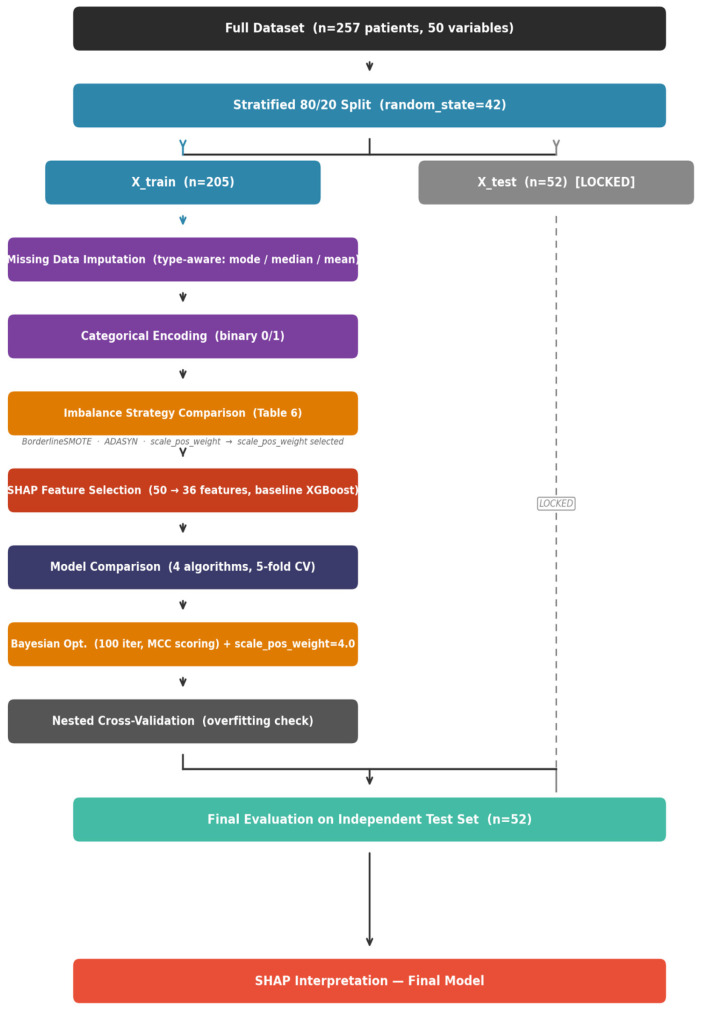
Data preprocessing workflow summarizing the main steps: stratified train/test split, handling missing data, encoding categorical variables, outlier treatment, and scaling strategies.

An overview of the preprocessing workflow applied to the dataset is illustrated in Figure 1.

### 2.5. Train/Test Split

To prevent data leakage, all preprocessing steps were applied strictly after the train/test split. The 257 patients were first divided into a training set (*n* = 205, 80%) and an independent test set (*n* = 52, 20%) using stratified random sampling (random_state = 42), preserving the outcome prevalence in both subsets (training: 37.1% deficient; test: 36.5% deficient). All subsequent preprocessing transformers were fitted exclusively on X_train and applied identically to X_test via sklearn pipeline.

### 2.6. Handling Missing Data

A type-aware imputation strategy was applied, fitted exclusively on X_train and applied to X_test via ColumnTransformer. Binary variables were imputed using mode (most frequent value); ordinal variables using median; continuous variables with approximately symmetric distributions using mean; and continuous variables with skewed distributions (|skewness| ≥ 1.0) using median. Post-imputation, binary and ordinal variables were rounded to restore their original discrete structure.

### 2.7. Encoding Categorical Variables

Binary categorical variables were encoded as 0/1 integers. Multi-category variables were expanded into binary indicator columns, with non-informative or redundant categories removed. Encoding was fitted on X_train and applied consistently to X_test to prevent category inconsistencies.

### 2.8. Handling Class Imbalance

The dataset exhibited moderate class imbalance, with 37.0% of patients classified as vitamin B12-deficient (*n* = 95) and 63.0% as non-deficient (*n* = 162). To address this, we initially compared generative over-sampling techniques (BorderlineSMOTE and ADASYN) against a native cost-sensitive learning approach using XGBoost’s scale_pos_weight hyperparameter. Although generative methods like BorderlineSMOTE achieved higher *MCC* (0.404 vs. 0.130) and Sensitivity (0.729 vs. 0.513) in cross-validation, they exhibited severe instability during Nested Cross-Validation and risk generating unrealistic synthetic patient profiles in small clinical datasets, potentially deceiving tree-based algorithms. Furthermore, applying resampling globally before cross-validation introduces fold contamination, compromising the validity of performance estimates. Consequently, the cost-sensitive approach was selected for the final pipeline. By optimizing scale_pos_weight dynamically within the Bayesian search space (optimized to 4.0), the model explicitly penalizes false negatives without altering the true distribution of the data. This strategy ensures robust generalization while maximizing the screening Sensitivity required for clinical safety.

### 2.9. Feature Selection

Feature selection was performed using SHAP (SHapley Additive exPlanations) values derived from the baseline XGBoost model trained on the full training set (*n* = 205). For each feature, the mean absolute SHAP value was computed across all training observations, quantifying its average contribution to the model’s predictions. Features with a mean absolute SHAP value strictly greater than zero were retained, yielding 36 features from the initial 50 variables. This threshold ensures that only features with demonstrable predictive contribution are included, without introducing arbitrary cutoffs. The top five features by mean absolute SHAP value were: HbA1c (1.052), Microalbuminuria (0.873), DN4 Score (0.663), Autonomic Neuropathy (0.655), and BMI (0.572). This SHAP-based selection approach replaces conventional filter or wrapper methods, offering consistency with the final model’s decision logic and avoiding the risk of selecting features optimized for a linear boundary rather than gradient boosting.

### 2.10. Model Development and Evaluation

#### 2.10.1. Model Training

Several classification algorithms were evaluated, including Logistic Regression (L2), Random Forest, Support Vector Machine (SVM), and XGBoost [16,17]. Each was assessed using a range of performance metrics, with particular emphasis on the Matthews correlation coefficient (*MCC*) due to its relevance for imbalanced datasets. XGBoost was selected for final development based on its superior post-optimization performance, its capacity to model nonlinear interactions, and its native compatibility with SHAP-based interpretability. An initial XGBoost model was trained on the full feature set using a default configuration with the evaluation metric set to logloss, serving as a performance baseline prior to optimization. Following SHAP-based feature selection (50 → 36 features), hyperparameter tuning was subsequently conducted using Bayesian optimization with stratified 5-fold cross-validation on the training set. The optimization process yielded the set of hyperparameters presented in Table 1. This optimized model was then evaluated on an independent test set to ensure an unbiased estimation of its predictive performance.

#### 2.10.2. Purpose of Hyperparameter OptimizationI

Bayesian hyperparameter optimization (BayesSearchCV, 100 iterations × 5-fold stratified cross-validation, scoring = *MCC*) served three explicit goals: (1) stabilize the model against overfitting through optimization of L1/L2 regularization parameters (reg_alpha, reg_lambda) and scale_pos_weight; (2) optimize the sensitivity–specificity trade-off for a clinical screening context, where missing a deficient patient (false negative) carries greater clinical cost than a false positive; and (3) identify the optimal tree structure (n_estimators = 108, max_depth = 2, learning_rate = 0.093). Compared to the baseline XGBoost model, optimization improved test AUC from 0.649 to 0.671 (+3.4%) and sensitivity from 0.316 to 0.737 (+133.2%), confirming that hyperparameter tuning meaningfully enhanced the model’s clinical utility beyond the default configuration.

#### 2.10.3. Performance Metrics

To thoroughly evaluate the predictive performance of the final model, several metrics suitable for imbalanced classification tasks were employed. The primary metric, *MCC*, provides an overall balanced assessment of classification accuracy. Additional metrics included balanced accuracy, sensitivity, specificity, ROC-AUC (area under the Receiver Operating Characteristic curve), and Brier Score. Sensitivity assessed the model’s capacity to identify true positive cases (patients with vitamin B12 deficiency), while specificity evaluated the correct identification of negative cases. Balanced accuracy, calculated as the average of sensitivity and specificity, provided an unbiased metric despite class imbalance. ROC-AUC offered comprehensive views of model performance across classification thresholds. The Brier Score assessed probabilistic calibration. All metrics were computed on the independent test set following hyperparameter optimization, ensuring a rigorous and unbiased evaluation of the model’s predictive capabilities.

#### 2.10.4. Mathematical Definitions of Evaluation Metrics

Model performance was assessed using the following metrics. The Matthews Correlation coefficient (*MCC*) was selected as the primary optimization metric, as it accounts for all four quadrants of the confusion matrix and is robust to class imbalance [18,19]:

$$MCC\;=\;(TP\;\times \;TN\;-\;FP\;\times \;FN)\;/\;\sqrt{\left[(TP+FP)(TP+FN)(TN+FP)(TN+FN)\right]}$$

where *TP*, *TN*, *FP*, and *FN* denote true positives, true negatives, false positives, and false negatives, respectively. Additional metrics were defined as follows:

- Sensitivity (Recall) = *TP*/(*TP* + *FN*).
- Specificity = *TN*/(*TN* + *FP*).
- Balanced Accuracy = (Sensitivity + Specificity)/2.
- ROC-AUC: area under the receiver operating characteristic curve, measuring discrimination across all classification thresholds.
- Brier Score = (1/n) ∑(p^i − yi)2, measuring calibration, where p^i is the predicted probability and *y*_i_ is the observed binary outcome.

An *MCC* of 1 indicates perfect prediction, 0 indicates no better than random, and −1 indicates total disagreement. Unlike accuracy or F1-score, *MCC* remains informative under class imbalance, making it the preferred primary metric for this study.

#### 2.10.5. Decision Threshold Optimization

The default classification threshold of 0.5 was replaced by an optimized threshold derived from the Youden Index (J = Sensitivity + Specificity − 1), which identifies the threshold maximizing the sum of sensitivity and specificity. To prevent data leakage, the optimal threshold was computed exclusively on out-of-fold (OOF) predictions from the training set and applied blindly to the independent test set. This approach follows the methodological recommendation of Fluss et al. [20] and is consistent with the pipeline leakage prevention strategy described above. The optimal threshold derived was 0.500, yielding a sensitivity of 0.737 and specificity of 0.545 on the independent test set.

#### 2.10.6. Justification of Model Complexity

To empirically justify the selection of XGBoost over simpler alternatives, four supervised learning models were compared on the training set under identical conditions (5-fold stratified cross-validation, no resampling, no hyperparameter optimization): Logistic Regression with L2 regularization, Random Forest, Support Vector Machine (SVM), and baseline XGBoost. Although Logistic Regression (L2) achieved the highest cross-validation *MCC* without optimization (0.264), optimized XGBoost exceeded this (*MCC* = 0.306 in CV) while achieving a comparable test AUC (0.671 vs. 0.685 for LR L2 in CV) and a substantially improved sensitivity (0.737 vs. 0.526). Model complexity was constrained via L1 regularization (reg_alpha = 1.0), L2 regularization (reg_lambda = 24.94), and a maximum tree depth of 2, preventing memorization of noise. The nonlinear gradient boosting architecture captures complex interactions between predictors—such as the combined effect of low HbA1c and prolonged metformin exposure—that cannot be modelled by Logistic Regression. Furthermore, XGBoost natively supports SHAP-based interpretability, enabling the direct clinical explanation of individual predictions.

#### 2.10.7. Model Interpretation

To ensure transparency and clinical interpretability, model interpretation was performed using SHAP. This method provides a consistent framework to quantify the contribution of each feature to individual predictions, allowing for a detailed understanding of the model’s decision-making process. SHAP values were computed for the final XGBoost model to identify and rank the most influential variables. These values also facilitate visualization through summary plots, which offer insights into the global importance of features and the direction of their effects. This interpretability supports clinical validation and enhances trust in the model’s predictions.

#### 2.10.8. Software and Reproducibility

All analyses were performed using Python 3.12.12. Key library versions are reported in Table 2. A fixed random seed (random_state = 42) was applied to all stochastic processes, including the train/test split, cross-validation, BayesSearchCV, BorderlineSMOTE, and SHAP computations. As described above, the dataset was divided using a stratified 80/20 split (random_state = 42), yielding a training set of 205 patients (prevalence: 37.1%) and an independent test set of 52 patients (prevalence: 36.5%).

Bayesian hyperparameter optimization was performed over 100 iterations with 5-fold inner cross-validation, optimizing *MCC*. The search space was defined as follows: n_estimators [100–500], max_depth [2–5], learning_rate [0.01–0.2, log-uniform], subsample [0.5–0.9], colsample_bytree [0.5–0.9], min_child_weight [3–10], gamma [0.5–5], reg_alpha [1–20], reg_lambda [5–30], scale_pos_weight [1–4].

The complete analysis notebook is provided as Supplementary Materials and is publicly available at https://doi.org/10.5281/zenodo.19006296.

## 3. Results

### 3.1. Patients’ Characteristics and Vitamin B12 Deficiency Prevalence

Patients’ characteristics are summarized in Table 3. The prevalence of vitamin B12 deficiency was 37%: Seventy-three patients (28.4%) had a vitamin B12 level < 150 pmol/L, and 22 patients (8.6%) had a borderline vitamin B12 level associated with hyperhomocysteinemia.

Comparative clinical and laboratory characteristics between patients with and without vitamin B12 deficiency are presented in Table 4. Variables with statistically significant differences (*p* < 0.05) included HbA1c, fasting glucose, sex, BMI, and microalbuminuria.

### 3.2. Model Selection

Four supervised learning models were compared on the training set using 5-fold stratified cross-validation without resampling (Table 5). Although Logistic Regression (L2) showed the highest cross-validation *MCC* without optimization (0.264), optimized XGBoost exceeded this (*MCC* = 0.306 in CV) while offering nonlinear interaction modelling and SHAP-based interpretability, empirically justifying its selection.

### 3.3. Resampling Strategy Comparison

Initial experiments on the training set compared generative resampling strategies (BorderlineSMOTE and ADASYN) against native cost-sensitive learning (scale_pos_weight) and a baseline with no resampling. As shown in Table 6, during standard 5-fold cross-validation, generative methods artificially inflated performance metrics. BorderlineSMOTE and ADASYN achieved apparent *MCCs* of 0.404 and 0.402, respectively, with Sensitivities appearing to reach up to 74.8%.

In contrast, the native cost-sensitive approach using a Bayesian-optimized scale_pos_weight (4.0) yielded more conservative metrics during this standard CV phase (*MCC* = 0.130, Sensitivity = 0.513), though it still significantly improved upon the unweighted baseline, which severely failed to detect positive cases (Sensitivity = 0.303).

However, subsequent rigorous evaluation using Nested Cross-Validation revealed that the high metrics of generative methods were a result of severe overfitting. The generation of synthetic minority samples caused the tree-based algorithms to learn overly rigid and clinically unrealistic decision boundaries, leading to instability on strictly unseen data. Consequently, despite the seemingly lower initial CV scores, the optimized cost-sensitive approach (scale_pos_weight = 4.0) was selected for the final predictive pipeline. It effectively penalizes false negatives without distorting the underlying clinical data distribution, ensuring the robust generalization required for a safe clinical screening tool.

### 3.4. Baseline Model Performance

As shown in Table 7, the baseline XGBoost model achieved an ROC-AUC of 0.649 and an *MCC* of 0.153 on the independent test set, with a sensitivity of 0.316 and a specificity of 0.818. These results motivated further model refinement through Bayesian hyperparameter optimization.

### 3.5. Optimized Model Performance

The optimized XGBoost model achieved an ROC-AUC of 0.671, *MCC* of 0.273, sensitivity of 0.737, and specificity of 0.545 on the independent test set (Table 8), using a decision threshold of 0.500 derived from the Youden index on out-of-fold training predictions. Calibration was assessed via the Brier Score (0.259). The Brier Score indicates limited probabilistic calibration, attributable to the scale_pos_weight = 4.0, which deliberately shifts predicted probabilities to maximize sensitivity. This model is therefore intended as a discriminative screening tool rather than a probability estimator; the calibration curve (Supplementary Materials Figure S2) should be consulted before any clinical probability interpretation.

### 3.6. Cross-Validation and Nested CV

Five-fold stratified cross-validation metrics on the training set are reported in Table 9 (mean ± SD with 95% CI). To assess hyperparameter optimization bias, a nested cross-validation was performed (outer 5-fold/inner BayesSearchCV, 30 iterations). The nested CV AUC (0.614 ± 0.050) exceeded the principal CV AUC (0.598 ± 0.070, Δ = +0.016), confirming the absence of significant HPO overfitting.

### 3.7. Model Interpretability Results

**Figure 2:**
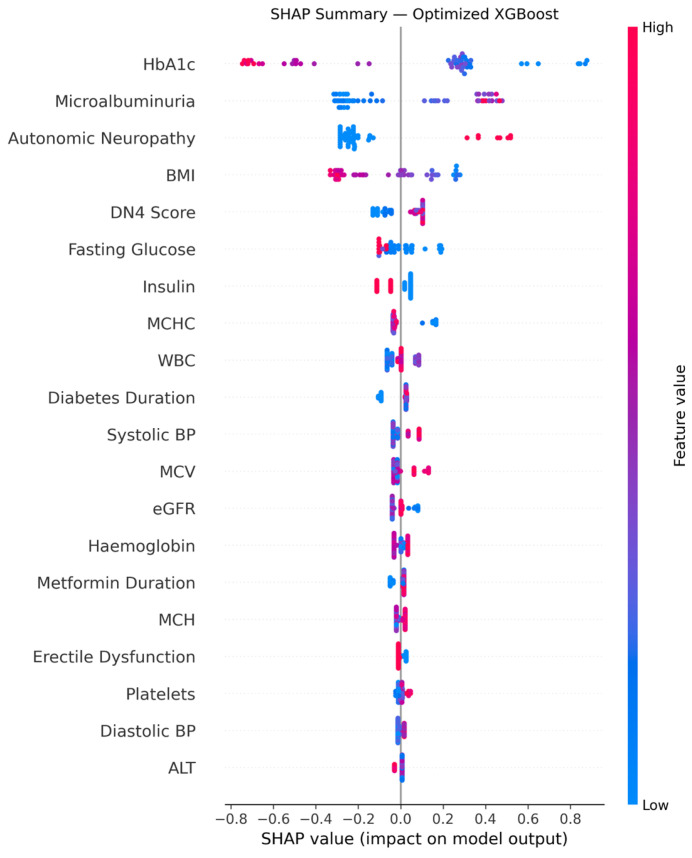
SHAP summary plot showing the relative importance and direction of influence of the top predictor variables in the XGBoost model for vitamin B12 deficiency prediction. ALT = Alanine Aminotransferase; BMI = body mass index; BP = blood pressure; eGFR = estimated glomerular filtration rate; MCH = Mean Corpuscular Hemoglobin; MCHC = Mean Corpuscular Hemoglobin Concentration; MCV = Mean Corpuscular Volume; WBCs = White Blood Cells.

The SHAP summary results are presented in Figure 2, which ranks input features by their overall impact on the XGBoost model’s predictions of vitamin B12 deficiency. HbA1c, microalbuminuria, autonomic neuropathy, BMI, DN4 score, and fasting glucose were identified as the most influential variables. Among these, higher HbA1c values were generally associated with a lower predicted probability of deficiency, while increased microalbuminuria levels were linked to a higher predicted risk. Autonomic neuropathy, BMI, DN4 score, and fasting glucose also contributed substantially to the model’s decisions.

### 3.8. Clinical Profile of Misclassified Cases

Table 10 presents the clinical profile of correctly and incorrectly classified patients based on the most influential SHAP features. Of the 52 test set patients, the model correctly classified 32 (18 *TN* + 14 *TP*) and misclassified 20 (5 *FN* + 15 *FP*), consistent with the test set sensitivity of 0.737 and specificity of 0.545.

False negatives (*FN*, *n* = 5) displayed a paradoxical combination of elevated HbA1c (10.08 ± 2.22) and DN4 scores (2.20 ± 1.79), mirroring the profile of true negatives (HbA1c = 10.01 ± 1.77, DN4 = 2.56 ± 2.33). This overlap suggests that the model relies heavily on elevated HbA1c as a signal against B12 deficiency—a clinically plausible association given the inverse relationship between glycemic control and B12 absorption—leading it to miss true deficits in patients lacking neuropathic symptoms. Additionally, *FN* patients had the highest BMI (35.07 ± 5.84), which may compound the misclassification risk, as obesity has been associated with lower circulating B12 levels independently of metformin use.

False positives (*FP*, *n* = 15) constituted the dominant error group, exhibiting moderate HbA1c (7.59 ± 0.86) and low DN4 scores (1.87 ± 2.03). The predominance of false positives over false negatives reflects the scale_pos_weight = 4 calibration and the Youden-derived threshold (0.500), which intentionally prioritizes sensitivity over specificity in this clinical screening context.

True negatives (*TN*, *n* = 18) displayed the highest HbA1c (10.01 ± 1.77) and the lowest microalbuminuria (10.29 ± 10.59 mg/L), reinforcing the model’s tendency to rule out deficiency in patients with poor glycemic control and preserved renal–metabolic profiles. Microalbuminuria showed wide variability across all groups—particularly in *FN* patients (33.58 ± 56.11)—limiting its individual discriminative utility in borderline cases.

**Figure 3:**
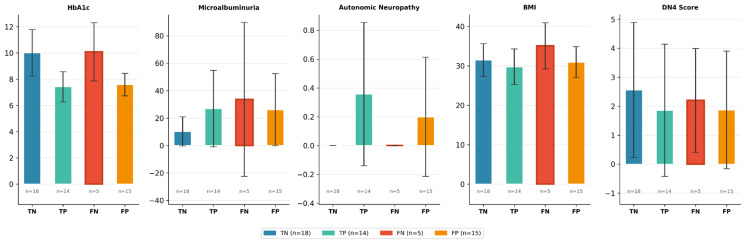
Bar plots (mean ± SD) of the five most influential SHAP features across classification groups (*TNs*: true negatives, *n* = 18; *TPs*: true positives, *n* = 14; *FNs*: false negatives, *n* = 5; *FPs*: false positives, *n* = 15). The *FN* group (red border) represents patients with true B12 deficiency missed by the model.

These findings, further supported by the distribution plots in Figure 3, illustrate that misclassifications predominantly arise when HbA1c and neuropathic/renal signals push in discordant directions, confirming the SHAP-identified feature hierarchy and highlighting the inherent difficulty of predicting B12 deficiency from clinical variables alone in a modest-sized cohort.

### 3.9. Nonlinear Interactions Captured by SHAP

Nonlinear interactions occur when the effect of one variable on the model’s prediction depends on the value of another variable, rather than being constant across all values as assumed in linear models. Gradient boosting algorithms such as XGBoost are particularly well-suited to capture such patterns because they build ensembles of decision trees that naturally model complex, conditional relationships between features. However, while these nonlinearities can improve predictive performance, they are often difficult to interpret directly from the model’s structure. SHAP addresses this by quantifying and visualizing how combinations of feature values jointly influence predictions.

Metformin treatment duration and cumulative dosage are well-established risk factors for vitamin B12 deficiency [1,2]. Their potential interaction with HbA1c in predicting B12 deficiency has been suspected. Therefore, we investigated the interaction between HbA1c and cumulative metformin dose using SHAP dependence plots.

**Figure 4:**
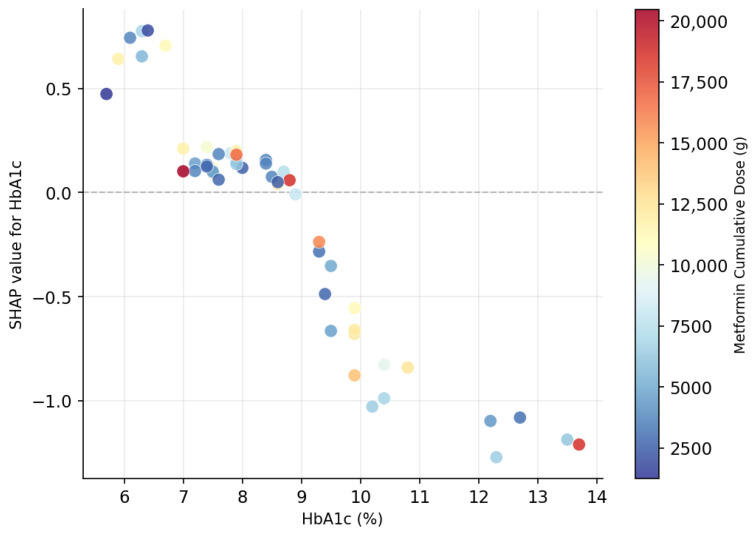
SHAP dependence plot for HbA1c, colored by cumulative metformin dose. Positive SHAP values indicate a contribution toward a higher predicted risk of B12 deficiency. Each point represents one test set patient (*n* = 52).

In Figure 4, we observe how the SHAP values for HbA1c vary as a function of glycemic control, colored by the cumulative metformin dose. When HbA1c is low (well-controlled diabetes), SHAP values tend to be positive, meaning that a low HbA1c contributes to a higher predicted risk of B12 deficiency. This counterintuitive effect is particularly pronounced when the cumulative metformin dose is also high (darker pink), suggesting that the model captures an indirect association where good glycemic control combined with prolonged metformin exposure could signal greater drug adherence and thus, higher risk of B12 depletion—a clinically plausible pattern consistent with long-term metformin effects.

## 4. Discussion

To the best of our knowledge, this is the first study to apply the XGBoost gradient boosting algorithm to predict vitamin B12 deficiency in patients with type 2 diabetes on metformin treatment. The dataset included 50 features selected through expert input and statistical filtering, and the target variable was redefined to incorporate both serum vitamin B12 and homocysteine levels, offering a more clinically relevant representation of deficiency. Feature selection was performed using a SHAP-based approach, retaining 36 features with non-zero mean absolute SHAP values, ensuring that only features with demonstrable predictive contribution were retained. The comparative analysis of four machine learning models—XGBoost, Random Forest, SVM, and Logistic Regression (L2)—enabled robust benchmarking, with XGBoost selected for its superior post-optimization performance, nonlinear modelling capacity, and native SHAP compatibility. To enhance interpretability, SHAP values were used to quantify individual feature contributions, providing transparent insights into the model’s decision-making process. This interpretable, data-driven workflow supports clinical reasoning and highlights the model’s potential for real-world integration, particularly within electronic medical records or as part of early screening strategies for patients with T2D at risk of vitamin B12 deficiency.

The final XGBoost model showed potential to distinguish between patients with and without vitamin B12 deficiency using routinely available clinical features, achieving a test set ROC-AUC of 0.671 [0.514–0.818] and an *MCC* of 0.273 [0.018–0.517]. Interpretability analysis revealed that the model’s predictions were primarily influenced by HbA1c, microalbuminuria, autonomic neuropathy, BMI, and DN4 score—all of which are readily available in clinical practice. Misclassification analysis highlighted a dominant pattern of false positives (*n* = 15) in patients with moderate HbA1c, while false negatives (*n* = 5) were characterised by elevated HbA1c and discordant neuropathic/renal signals. SHAP interaction plots further uncovered a nonlinear effect whereby low HbA1c combined with a high cumulative metformin dose was associated with increased predicted deficiency risk, consistent with the hypothesis that good glycemic control may reflect greater drug adherence and prolonged metformin exposure. These findings confirm that meaningful patterns can be extracted from structured clinical data without requiring invasive or costly diagnostic procedures and demonstrate that machine learning models—when guided by clinically relevant variables—can assist in the early identification of patients at risk.

### 4.1. Vitamin B12 Status, Glycemic Control and Chronic Exposure to Metformin

The relationship between vitamin B12 status and glycemic control is poorly elucidated. The findings of various studies conducted on this topic are inconsistent [21,22,23,24,25]. Some studies conducted in non-diabetic or pre-diabetic individuals have found that vitamin B12 deficiency was associated with increased HbA1c levels [25,26]. These findings have been attributed to a prolonged erythrocyte lifespan in situations of vitamin B12 deficiency. This phenomenon has been more extensively demonstrated in individuals with iron deficiency [27,28,29].

In patients with diabetes, the relationship between vitamin B12 and HbA1c has been less studied, and findings are more inconsistent [21,22,23,24,25,30,31]. In the present study, HbA1c emerged as the strongest predictive factor for vitamin B12 deficiency. Lower HbA1c levels were associated with an increased predicted probability of vitamin B12 deficiency, whereas higher levels were associated with a reduced predicted risk. Similar findings have been reported in other studies employing statistical methods [23,29,30]. Fasting blood glucose also emerged as a potential factor influencing the prediction of vitamin B12 deficiency. This finding argues against assay interference affecting HbA1c measurements and instead supports the hypothesis of a direct impact of glycemic control. These unexpected results may be explained by different hypotheses.

The first hypothesis is that poorly controlled patients may have a masked vitamin B12 deficiency. Indeed, it has been demonstrated that, in the context of chronic hyperglycemia, vitamin B12 may be sequestered in the bloodstream, leading to a functional intracellular deficiency [22,23,25,32]. This phenomenon may result from an overload of advanced glycation end (AGE) products in patients with uncontrolled diabetes [22]. AGE overload may saturate megalin, which mediates the renal uptake of the transcobalamin–vitamin B12 complex. Thus, vitamin B 12 may be retained in the blood. As a result, circulating levels of vitamin B12 appear elevated or normal, while actual tissue availability may be reduced, leading to a state of functional vitamin B12 deficiency [22]. Glycation of transcobalamin or vitamin B12 receptors may also explain this phenomenon [22,23]. However, this hypothesis appears less likely in the present study, as the definition of vitamin B12 deficiency included borderline cases with elevated homocysteine levels, unlike in previous studies. This approach reduces the likelihood of overlooking functional intracellular deficiency.

A second hypothesis is that lower HbA1c reflects better treatment adherence, leading to higher cumulative metformin exposure and, consequently, an increased risk of metformin-induced vitamin B12 deficiency. This hypothesis is supported by the fact that the model captured an indirect association between HbA1c and cumulative metformin dose. Thus, good glycemic control when combined with prolonged metformin exposure predicts a higher risk of vitamin B12 depletion.

The potential link between glycemic control and vitamin B12 deficiency warrants further investigation, as it could have implications for the management of T2D.

### 4.2. Vitamin B12 Status and Renal Function

Renal impairment may influence serum vitamin B12 concentrations. In the present study, increased microalbuminuria and reduced eGFR were linked to a higher predicted probability of vitamin B12 deficiency. The impact of chronic kidney disease (CKD) on vitamin B12 metabolism is controversial. Conflicting results have been reported. Some studies have shown that a significant proportion of patients with CKD exhibit low serum vitamin B12 levels [33,34,35]. Vitamin B12 deficiency in individuals with renal impairment may be due to malabsorption and dietary restrictions [36].

Opposite findings have also been reported. Abnormally elevated serum vitamin B12 concentrations were observed in some patients with renal failure [37,38,39]. Impaired renal function can lead to the accumulation of B12 binding proteins, resulting in artificially elevated serum vitamin B12 levels despite possible intracellular depletion [22]. Even when total vitamin B12 levels are high, as observed in uncontrolled diabetes, patients with CKD may experience a functional vitamin B12 deficiency due to impaired tissue uptake. Moreover, homocysteine—a metabolite dependent on vitamin B12—can be elevated in patients with CKD despite normal serum vitamin B12 levels, further indicating a potential functional deficiency [38].

### 4.3. Vitamin B12 Status and Diabetic Neuropathy

The link between vitamin B12 status and neurological disorders is well established. In the present study, both autonomic neuropathy and a high DN4 score—indicative of diabetic peripheral neuropathy—were identified as significant predictors of vitamin B12 deficiency. These results align with several studies and meta-analyses demonstrating that B12 deficiency is associated with an increased risk of developing or exacerbating diabetic neuropathy [40,41,42]. Furthermore, interventional research has shown that methylcobalamin administration can improve neurophysiological parameters [43]. Notably, autonomic neuropathy emerged as the third most influential predictor in our model (Rank 3, mean |SHAP| = 0.270), surpassing both BMI and the DN4 score. This finding underscores a potent link between vitamin B12 levels and autonomic nervous system integrity. Biologically, B12 deficiency impairs myelin synthesis and axonal maintenance, leading to both peripheral and autonomic fiber damage. The prominent predictive weight assigned to autonomic neuropathy by the model suggests that clinicians should consider systematic B12 screening for patients presenting with autonomic symptoms, even when overt peripheral neuropathy is absent.

### 4.4. Other Contributing Factors

In this study, BMI also appeared as a significant factor influencing the predictive model for vitamin B12 deficiency. Lower vitamin B12 levels have been associated with higher BMI and obesity [44,45,46]. However, this relationship is not always consistent across all studies and populations. As in this study, some authors demonstrated that vitamin B12 levels were lower among individuals with lower BMI [47]. Other studies have found no evidence of this association [48]. These inconsistencies can be attributed to the variability among study populations regarding age, ethnicity, socioeconomic status, and dietary patterns.

Advanced age is also a classic factor associated with vitamin B12 deficiency [49]. In this study, age did not emerge as a significant factor influencing the prediction model. This may be related to the relatively older study population, with a mean age of 60 years.

Duration of metformin treatment is a well-established factor associated with vitamin B12 deficiency [1,2]. Metformin duration emerged in our prediction model (rank 15, mean |SHAP| = 0.022), with a modest direct contribution. Notably, the SHAP interaction plot revealed that when metformin duration is combined with low HbA1c levels, the predicted probability of vitamin B12 deficiency increases substantially. This nonlinear interaction suggests that good glycemic control combined with prolonged metformin exposure reflects a “true” sustained drug exposure likely driven by better treatment adherence and carries a stronger predictive signal than either variable considered individually. This highlights the advantage of XGBoost in capturing complex interactions that linear models cannot detect.

Other risk factors reported in the literature, including chronic use of gastric antisecretory medications [50] and the presence of diabetic retinopathy [51], did not significantly affect the predictive probability of vitamin B12 deficiency in this study.

Finally, hematological parameters and anemia do not appear to have a significant impact on the prediction of vitamin B12 deficiency in patients with T2D treated with metformin. The MASTERMIND study and other research indicate that patients with T2D treated with metformin have a higher risk of anemia compared to those on a diet or other antihyperglycemic treatment [52,53]. Although anemia incidence increases with metformin treatment duration and dose, this risk does not correlate with vitamin B12 status [52,54]. These findings suggest that factors other than vitamin B12 deficiency contribute to anemia in metformin-treated patients with T2D.

### 4.5. Limitations

This study presents limitations that must be acknowledged. First, the relatively small sample size significantly constrains the model’s ability to capture complex clinical interactions and undermines the generalizability of the predictions. Moreover, despite extensive hyperparameter tuning and comparative evaluation of multiple supervised learning algorithms, none of the tested models exceeded a Matthews correlation coefficient of 0.310 on the independent test set. This performance ceiling highlights the inherent difficulty of modeling a multifactorial and imbalanced clinical outcome from a limited dataset. This study represents a single-centre proof-of-concept investigation, and the findings should be interpreted accordingly. External multi-centre validation on independent cohorts is explicitly recommended as the first research priority before any clinical deployment of this model.

### 4.6. Overfitting Mitigation Strategy

Despite the limited sample size, several complementary measures were implemented to mitigate overfitting. First, stratified 5-fold cross-validation was applied on the training set, with performance averaged across all folds (ROC-AUC = 0.598 ± 0.070), reducing selection bias from a single train/test split. Second, SHAP-based feature selection retained only the 36 features with mean |SHAP| > 0 from the initial 50 variables, improving the observations-to-variables ratio and reducing model complexity. Third, XGBoost regularization parameters (reg_alpha = 1.0, reg_lambda = 24.94, max_depth = 2) explicitly penalize model complexity, preventing memorization of noise patterns. Fourth, class imbalance was handled via the scale_pos_weight hyperparameter (optimized to 4.0 by BayesSearchCV), which penalizes false negatives without generating synthetic samples or risking fold contamination. Finally, a nested cross-validation (outer 5-fold/inner BayesSearchCV, 30 iterations) confirmed minimal hyperparameter optimization bias, with a nested AUC of 0.614 ± 0.050 versus a principal CV AUC of 0.598 ± 0.070 (Δ = +0.016), confirming the absence of significant HPO overfitting.

### 4.7. Nested Cross-Validation

To further assess the robustness of the hyperparameter optimization procedure, a nested cross-validation was performed (outer 5-fold/inner BayesSearchCV, 30 iterations). The nested CV yielded a mean ROC-AUC of 0.614 ± 0.050 and a mean *MCC* of 0.112 ± 0.076, compared to 0.598 ± 0.070 and 0.306 ± 0.069 in the principal cross-validation, respectively. The nested AUC slightly exceeded the principal CV estimate (ΔAUC = +0.016), while the *MCC* gap (Δ*MCC* = −0.194) reflects the threshold difference between the two schemes (Youden 0.500 in the main pipeline vs. fixed 0.5 in nested CV). These results confirm that hyperparameter optimization introduced minimal overfitting bias. The wider variance observed in the nested scheme reflects the reduced inner fold size inherent to double cross-validation and is expected given the modest sample size of 205 training patients.

## 5. Conclusions

This study presents, for the first time, the application of XGBoost to predict vitamin B12 deficiency in patients with T2D treated with metformin, using routinely available clinical and laboratory data. The model, supported by SHAP-based interpretation, identified HbA1c, microalbuminuria, autonomic neuropathy, BMI, DN4 score, and fasting glucose as key predictors, revealing clinically plausible risk patterns. Nonlinear SHAP interaction plots revealed an increased predicted risk in patients with low HbA1c combined with prolonged metformin exposure, consistent with better treatment adherence driving cumulative B12 depletion. Glycemic control combined with long-term metformin exposure, autonomic neuropathy, diabetic peripheral neuropathy, and renal impairment are thus the main predictive factors for vitamin B12 deficiency in patients with T2D treated with metformin. Despite the limitations of a small retrospective dataset, the approach achieved balanced performance with high sensitivity (0.737), highlighting its potential as a decision-support tool to optimize early detection and guide targeted screening in diabetes care.

## Figures and Tables

**Table 1:** Optimized hyperparameters for the XGBoost model derived from BayesSearchCV (100 iterations × 5-fold stratified cross-validation).

Hyperparameter	Value
colsample_bytree	0.9
gamma	2.866
learning_rate	0.093
max_depth	2
min_child_weight	10
n_estimators	108
reg_alpha	1.000
reg_lambda	24.940
scale_pos_weight	4.000
subsample	0.900

**Table 2:** Python library versions used in this study.

Library	Version	Role
Python	3.12.12	Base language
pandas	2.2.2	Data management
numpy	2.0.2	Numerical computing
scikit-learn	1.6.1	Preprocessing, evaluation
xgboost	3.2.0	Gradient boosting classifier
imbalanced-learn	0.14.1	BorderlineSMOTE
scikit-optimize	0.10.2	Bayesian hyperparameter search
shap	0.51.0	SHAP explainability
matplotlib	3.10.0	Visualisation
scipy	1.16.3	Statistical tests

**Table 3:** Patient’s characteristics (*n* = 257).

Variables	Value
Age, years	59.8 ± 7.9
Men, n (%)	121 (47.1)
Diabetes duration, years	10.4 ± 5.3
Cardiovascular disease, n (%)	56 (21.8)
Use of acid-lowering agents, n (%)	90 (35.0)
Body mass index, kg/m^2^	30.4 ± 4.8
HbA1c, %	8.7 ± 1.9
Daily dose of metformin, mg	2072 ± 540
Metformin treatment duration, years	10.2 ± 5.2
Insulin treatment, n (%)	90 (35.0)

**Table 4:** Comparative clinical and laboratory characteristics by vitamin B12 deficiency status.

Variable	Non-Deficient (*n* = 162)	Deficient (*n* = 95)	*p*-Value
Sociodemographic and clinical characteristics			
Sex, male n (%)	65 (40.1%)	56 (58.9%)	0.005
Age, years—median (IQR)	61.0 (54.3–65.0)	60.0 (56.0–65.0)	0.741
Diabetes Duration, years	9.0 (6.0–14.5)	10.0 (7.0–15.0)	0.383
Hypertension	61 (64.2%)	96 (59.3%)	0.514
BMI, kg/m^2^—median (IQR)	30.73 (27.85–33.69)	29.62 (26.75–31.69)	0.021
Diabetic Retinopathy, n (%)	39 (24.8%)	20 (21.7%)	0.688
DN4 Score—median (IQR)	1.50 (0.00–4.00)	2.00 (0.00–4.00)	0.354
Peripheral neuropathy, n (%)	95 (58.6%)	45 (47.4%)	0.105
Autonomic neuropathy, n (%)	36 (22.2%)	41 (43.2%)	0.001
Macroangiopathy, n (%)	32 (19.8%)	24 (25.3%)	0.381
Biological characteristics			
HbA1c, %—median (IQR)	8.75 (7.62–10.10)	7.90 (6.70–8.95)	<0.001
Fasting Glucose, mmol/L	1.93 (1.56–2.94)	1.75 (1.42–2.33)	0.012
eGFR, mL/min/1.73 m^2^	89.65 (78.06–92.80)	88.02 (72.78–94.10)	0.199
Microalbuminuria, mg/24 h	10.98 (5.03–30.38)	21.00 (6.70–60.80)	0.009
Haemoglobin, g/dL	13.20 (12.45–14.00)	13.20 (12.20–14.38)	0.880
MCV, fL	85.20 (81.62–87.78)	86.20 (82.35–89.17)	0.173
AST, U/L	16.0 (14.0–20.0)	16.0 (14.0–19.0)	0.354
ALT, U/L	17.0 (14.0–26.0)	17.0 (13.5–21.0)	0.112
Treatment			
Metformin Duration, years	9.0 (6.0–13.0)	10.0 (6.5–14.5)	0.410
Metformin Cumulative Dose, g	6205.0 (3723.0–10,238.3)	7446.0 (4343.5–10,858.8)	0.341
Sulfonylureas, n (%)	78 (48.1%)	52 (54.7%)	0.373
Insulin, n (%)	62 (38.3%)	28 (29.5%)	0.196
Proton Pump Inhibitor, n (%)	59 (36.4%)	31 (32.6%)	0.632

*Data are expressed as median (IQR) for continuous variables or n (%) for categorical variables. Mann–Whitney U test used for non-normal continuous variables; independent t-test for normally distributed variables; Chi-square or Fisher’s exact test for categorical variables. Highlighted rows indicate statistically significant differences (p < 0.05). B12 deficiency is defined as serum B12 < 150 pmol/L or borderline (150–221 pmol/L) with hyperhomocysteinaemia > 15 μmol/L.*

**Table 5:** Performance comparison of four supervised learning models on the training set (5-fold stratified cross-validation without resampling).

Model	AUC	*MCC*	Sensitivity	Specificity
Logistic Regression (L2)	0.685	0.264	0.526	0.736
SVM	0.595	−0.123	0.303	0.574
XGBoost (baseline)	0.610	0.076	0.368	0.705
Random Forest	0.591	0.049	0.184	0.853
XGBoost (optimised)	0.671 *	0.273 *	0.737 *	0.545 *

* Independent test set metrics after BayesSearchCV optimization (100 iterations).

**Table 6:** Comparison of resampling strategies on the training set (5-fold stratified CV, baseline XGBoost).

Strategy	*MCC*	Balanced Accuracy	Sensitivity	Specificity
No Resampling	0.025	0.512	0.303	0.721
scale_pos_weight = 1.70 (natural ratio)	0.101	0.550	0.434	0.667
scale_pos_weight = 4.0 (optimized)	0.130	0.567	0.513	0.620
ADASYN	0.402	0.700	0.748	0.651

**Table 7:** Baseline XGBoost performance on the independent test set.

Metric	Value
ROC-AUC	0.649
*MCC*	0.153
Sensitivity	0.316
Specificity	0.818

**Table 8:** Optimized XGBoost performance on the independent test set with bootstrap 95% confidence intervals (2000 resamples, *n* = 52).

Metric	Value	Bootstrap 95% CI
ROC-AUC	0.671	[0.514–0.818]
*MCC*	0.273	[0.018–0.517]
Balanced Accuracy	0.641	[0.510–0.770]
Sensitivity	0.737	[0.533–0.938]
Specificity	0.545	[0.375–0.710]
Brier Score	0.259	—
Decision Threshold (Youden)	0.500471	—

*Bootstrap 95% CI derived from 2000 resamples on the independent test set (n = 52).*

**Table 9:** Cross-validation metrics (OOF predictions, training set) and nested CV comparison.

Metric	CV Mean ± SD	CV 95% CI	Nested CV Mean ± SD	Δ
ROC-AUC	0.598 ± 0.070	[0.513–0.677]	0.614 ± 0.050	+0.016
*MCC*	0.306 ± 0.069	[0.215–0.371]	0.112 ± 0.076	−0.194
Sensitivity	0.962 ± 0.056	[0.881–1.000]	0.711 ± 0.152	−0.251 *
Specificity	0.287 ± 0.049	[0.235–0.355]	0.396 ± 0.133	+0.109
Bal. Accuracy	0.625 ± 0.027	[0.586–0.652]	—	—

* Sensitivity difference reflects threshold difference (Youden 0.500 in main pipeline vs. fixed 0.5 in nested CV), not HPO bias.

**Table 10:** Clinical profile of correctly and incorrectly classified patients based on the most influential SHAP features.

Group	n	HbA1c (%) Means ± SD	Microalbuminuria (mg/L) Means ± SD	Autonomic Neuropathy Means ± SD	BMI (kg/m^2^) Means ± SD	DN4 Score (/10) Means ± SD
True Negatives (*TNs*)	18	10.01 ± 1.77	10.29 ± 10.59	0.00 ± 0.00	31.49 ± 4.12	2.56 ± 2.33
True Positives (*TPs*)	14	7.42 ± 1.15	26.87 ± 27.78	0.36 ± 0.50	29.74 ± 4.53	1.86 ± 2.28
False Negatives (*FNs*)	5	10.08 ± 2.22	33.58 ± 56.11	0.00 ± 0.00	35.07 ± 5.84	2.20 ± 1.79
False Positives (*FPs*)	15	7.59 ± 0.86	26.08 ± 26.19	0.20 ± 0.41	30.95 ± 3.92	1.87 ± 2.03

*Data presented as mean ± SD. TNs: true negatives; TPs: true positives; FNs: false negatives; FPs: false positives. Highlighted row (FN) indicates cases where the model failed to detect true B12 deficiency.*

## Data Availability

The original contributions presented in this study are included in the article/Supplementary Material. Further inquiries can be directed to the corresponding author. The analysis code is openly available on Zenodo (https://doi.org/10.5281/zenodo.19006296).

## Supplementary Materials

The following supporting information can be downloaded at: https://www.mdpi.com/article/10.3390/metabo16040227/s1.

## Abbreviations

The following abbreviations are used in this manuscript:

BMI	Body mass index
T2D	Type 2 diabetes
DN4	Neuropathic Pain 4 questions
*MCC*	Matthews correlation coefficient
SHAP	SHapley Additive exPlanations
XGBoost	eXtreme Gradient Boosting
