# Supplementary material for: Interpretable Machine Learning to Predict Metformin-Induced Vitamin B12 Deficiency: Association with Glycemic Control and Neuropathic Symptoms
Source: Metabolites. 2026 Mar 30;16(4):227. doi: 10.3390/metabo16040227 (PMC13118126; doi:10.3390/metabo16040227)
Supplement: Supplementary file 1 [file metabolites-16-00227-s001.zip › Supplemental Material/B12_Supplementary_S2_with_code.html]

B12\_Supplementary\_S2\_final


# Supplementary Material S2 — Analysis Notebook¶

## Interpretable Machine Learning to Predict Metformin-Induced Vitamin B12 Deficiency: Association with Glycemic Control and Neuropathic Symptoms¶

**Authors:** Yassmin Salhi, Meriem Yazidi, Amine Dhraief, Elyes Kammoun, Melika Chihaoui, Tamim Alsuliman, Layth Sliman

**Corresponding author:** Meriem Yazidi — meriem.yazidi@fmt.utm.tn

**Journal:** *(under review)*

**Date:** March 2026

---

## Description¶

This notebook provides the complete, fully reproducible analysis pipeline for the above manuscript. It covers:

1. **Data loading and preprocessing** — type-aware imputation (median/mode), column renaming (French → English)
2. **Exploratory analysis** — B12 deficiency distribution, missing data summary, library versions
3. **SHAP-based feature selection** — baseline XGBoost trained on full training set; 36 features with mean |SHAP| > 0 retained from 50 initial variables
4. **Class imbalance comparison** — five strategies evaluated: BorderlineSMOTE, ADASYN, No Resampling, scale\_pos\_weight=1.70, scale\_pos\_weight=4.0
5. **Model comparison** — XGBoost vs Logistic Regression (L2), Random Forest, SVM on 5-fold stratified CV (Table 5)
6. **Bayesian hyperparameter optimization** — BayesSearchCV, 100 iterations × 5-fold CV, scoring = MCC
7. **Final evaluation** — test set metrics, Youden threshold, calibration curve, bootstrap 95% CIs (2000 resamples)
8. **Nested CV** — outer 5-fold / inner BayesSearchCV (30 iterations) to assess HPO bias
9. **SHAP interpretability** — summary plot, beeswarm, waterfall, dependence plot (HbA1c × cumulative metformin dose)
10. **Misclassification analysis** — clinical profiles of FN, FP, TP, TN cases

---

## Reproducibility¶

| Parameter | Value |
| --- | --- |
| Random seed | `random_state = 42` (all stochastic steps) |
| Train/test split | Stratified 80/20 — Train: n=205, Test: n=52 |
| Python | 3.12.12 |
| XGBoost | 3.2.0 |
| SHAP | 0.51.0 |
| scikit-learn | 1.6.1 |
| scikit-optimize | 0.10.2 |
| imbalanced-learn | 0.14.1 |

See **Cell 6** for the full library version table.

---

## Data Availability¶

The dataset is not publicly available due to patient confidentiality constraints (La Rabta University Hospital Ethics Committee). Access may be requested from the corresponding author subject to a data sharing agreement.

---

> **To run this notebook:** Upload the dataset file when prompted in Cell 8 (Google Colab), then run all cells in order (`Runtime → Run all`).

# 🧬 B12 Deficiency Prediction — XGBoost Pipeline¶

**Interpretable Machine Learning to Predict Metformin-Induced Vitamin B12 Deficiency**

---

| Section | Description |
| --- | --- |
| 0 | Setup & Configuration |
| 1 | Data Loading & Cleaning |
| 2 | Exploratory Data Analysis |
| 3 | Preprocessing |
| 4 | Resampling Strategy Selection |
| 5 | Model Selection & SHAP Feature Selection |
| 6 | Hyperparameter Optimization & Evaluation |
| 7 | Interpretability & Publication Figures |

## ⚙️ Section 0 — Setup & Configuration¶

> Google Drive connection, imports, global configuration (seed, paths, figure quality).

In [1]:

```
# ─────────────────────────────────────────────────────────────
# Install missing dependencies
# ─────────────────────────────────────────────────────────────
!pip install scikit-optimize --quiet
print('Dependencies installed ✅')
```

```
   ━━━━━━━━━━━━━━━━━━━━━━━━━━━━━━━━━━━━━━━━ 0.0/107.8 kB ? eta -:--:--
   ━━━━━━━━━━━━━━━━━━━━━━━━━━━━━━━━━━━━━━━━ 107.8/107.8 kB 3.7 MB/s eta 0:00:00
Dependencies installed ✅
```

In [2]:

```
# ─────────────────────────────────────────────────────────────
# Imports
# ─────────────────────────────────────────────────────────────
import os
import time
import warnings
warnings.filterwarnings('ignore')

import pandas as pd
import numpy as np
import seaborn as sns
import matplotlib.pyplot as plt
from scipy import stats

from sklearn.model_selection import (
    train_test_split, StratifiedKFold,
    cross_val_predict, cross_validate
)
from sklearn.compose import ColumnTransformer
from sklearn.pipeline import Pipeline
from sklearn.impute import SimpleImputer
from sklearn.linear_model import LogisticRegression
from sklearn.ensemble import RandomForestClassifier
from sklearn.svm import SVC
from sklearn.metrics import (
    confusion_matrix, roc_auc_score, roc_curve,
    accuracy_score, balanced_accuracy_score,
    matthews_corrcoef, recall_score, precision_score,
    average_precision_score, f1_score, brier_score_loss,
    classification_report, make_scorer
)
from sklearn.calibration import calibration_curve

import xgboost as xgb
from xgboost import XGBClassifier

from imblearn.pipeline import Pipeline as ImbPipeline
from imblearn.over_sampling import BorderlineSMOTE, ADASYN

from skopt import BayesSearchCV
from skopt.space import Integer, Real, Categorical

import shap
```

In [3]:

```
# ─────────────────────────────────────────────────────────────
# Global configuration
# ─────────────────────────────────────────────────────────────

# Reproducibility seed
SEED = 42
np.random.seed(SEED)

# ── Output paths (local) ─────────────────────────────────────
FIGURES_PATH = 'outputs/figures'
TABLES_PATH  = 'outputs/tables'

os.makedirs(FIGURES_PATH, exist_ok=True)
os.makedirs(TABLES_PATH,  exist_ok=True)

print('Figures  →', FIGURES_PATH)
print('Tables   →', TABLES_PATH)

# ── Global figure quality settings ───────────────────────────
plt.rcParams.update({
    'figure.dpi'     : 150,
    'savefig.dpi'    : 300,
    'savefig.bbox'   : 'tight',
    'font.size'      : 11,
    'axes.titlesize' : 13,
    'axes.labelsize' : 12,
    'legend.fontsize': 10,
    'font.family'    : 'DejaVu Sans',
})

SAVEFIG_KW = dict(dpi=300, bbox_inches='tight')
```

```
Figures  → outputs/figures
Tables   → outputs/tables
```

In [4]:

```
# ─────────────────────────────────────────────────────────────
# Library versions (reproducibility)
# ─────────────────────────────────────────────────────────────
import sys, sklearn, imblearn, skopt
import matplotlib, scipy

def get_version(module):
    try:
        return module.__version__
    except AttributeError:
        return 'Unknown'

versions = {
    'Python'          : sys.version.split()[0],
    'pandas'          : get_version(pd),
    'numpy'           : get_version(np),
    'seaborn'         : get_version(sns),
    'matplotlib' : matplotlib.__version__,
    'scipy'      : scipy.__version__,
    'scikit-learn'    : get_version(sklearn),
    'xgboost'         : get_version(xgb),
    'shap'            : get_version(shap),
    'imbalanced-learn': get_version(imblearn),
    'scikit-optimize' : get_version(skopt),


}

print(f"{'Library':<20} {'Version'}")
print('-' * 35)
for lib, ver in versions.items():
    print(f"{lib:<20} {ver}")
```

```
Library              Version
-----------------------------------
Python               3.12.12
pandas               2.2.2
numpy                2.0.2
seaborn              0.13.2
matplotlib           3.10.0
scipy                1.16.3
scikit-learn         1.6.1
xgboost              3.2.0
shap                 0.51.0
imbalanced-learn     0.14.1
scikit-optimize      0.10.2
```

---

## 🗂️ Section 1 — Data Loading & Cleaning¶

> CSV loading, removal of irrelevant columns, error code handling (9, 99...) and binary variable conversion.

In [5]:

```
# ─────────────────────────────────────────────────────────────
# Data Loading
# ─────────────────────────────────────────────────────────────
# ⚠️  DATA AVAILABILITY NOTICE:
# The dataset used in this study contains identifiable patient
# information collected at La Rabta University Hospital, Tunis,
# Tunisia (September 2019 – June 2020) and is not publicly
# available due to ethical and patient confidentiality constraints.
# Access to anonymised data may be requested from the corresponding
# author (meriem.yazidi@fmt.utm.tn) subject to approval by the Ethics
# Committee of La Rabta University Hospital and signature of a
# data sharing agreement.
# ─────────────────────────────────────────────────────────────
from google.colab import files
uploaded = files.upload()

df = pd.read_csv('IA-B12-26-2-26.csv', sep=';', decimal=',')
print(f'Raw dataset: {df.shape[0]} patients, {df.shape[1]} variables')

# ── Column renaming — French clinical labels → English ───────
COLUMN_RENAME = {
    # Demographics
    'genre'                                : 'Sex',
    'Age'                                  : 'Age',
    'BMI'                                  : 'BMI',
    'ATCDanemie'                           : 'History of Anaemia',
    'HTA'                                  : 'Hypertension',
    'coronaropathies'                      : 'Coronary Artery Disease',
    'AVCouAIT'                             : 'Stroke or TIA',
    'AOMI'                                 : 'Peripheral Artery Disease',
    'MACROANGIOPATHIES'                    : 'Macroangiopathy',
    'signespsy'                            : 'Psychiatric Signs',
    'Anciennetédiabete'                    : 'Diabetes Duration',
    'Dureemetformine'                      : 'Metformin Duration',
    'Mgparjour'                            : 'Metformin Daily Dose',
    'DoseCumulee'                          : 'Metformin Cumulative Dose',
    'Sulfamides'                           : 'Sulfonylureas',
    'Insuline'                             : 'Insulin',
    'sous_antisecretoire_gastrique'        : 'Proton Pump Inhibitor',
    'OBSERVANCE'                           : 'Treatment Adherence',
    'IUM'                                  : 'Urinary Infection',
    'Retinopathie'                         : 'Diabetic Retinopathy',
    'Nephropathiediabetique'               : 'Diabetic Nephropathy',
    'dysfonctionerectile'                  : 'Erectile Dysfunction',
    'Neuropathieperipherique'              : 'Peripheral Neuropathy',
    'MICROANGIOPATHIES'                    : 'Microangiopathy',
    'ScoreDN4'                             : 'DN4 Score',
    'ScoreNISreflexes'                     : 'NIS Reflexes Score',
    'ScoreNISsensibilité'                  : 'NIS Sensitivity Score',
    'scoreNIStotal'                        : 'NIS Total Score',
    'scoregirerd'                          : 'Girerd Score',
    'TAScouché'                            : 'Systolic BP',
    'TADcouché'                            : 'Diastolic BP',
    'TauxHb'                               : 'Haemoglobin',
    'VGM'                                  : 'MCV',
    'TCMH'                                 : 'MCH',
    'CCMH'                                 : 'MCHC',
    'GB'                                   : 'WBC',
    'Plaquettes'                           : 'Platelets',
    'ANEMIE'                               : 'Anaemia',
    'hypochromie'                          : 'Hypochromia',
    'macrocytose'                          : 'Macrocytosis',
    'leucopenie'                           : 'Leucopenia',
    'thrombopenie'                         : 'Thrombocytopenia',
    'GAJ'                                  : 'Fasting Glucose',
    'HBA1c'                                : 'HbA1c',
    'Clairancecreatinine'                  : 'eGFR',
    'microalbuminurie'                     : 'Microalbuminuria',
    'ASAT'                                 : 'AST',
    'ALAT'                                 : 'ALT',
    'neuropathievegetative'                : 'Autonomic Neuropathy',
    'Carence_ou_borderline_avec_hyperhcy'  : 'B12_Deficiency',
}

df = df.rename(columns=COLUMN_RENAME)

assert 'B12_Deficiency' in df.columns, \
    'ERROR: B12_Deficiency column not found after renaming. Check CSV separator and column names.'

print(f'Dataset loaded and renamed: {df.shape[0]} patients, {df.shape[1]} variables')
print(f'Target column confirmed: B12_Deficiency ✅')
df.head()
```

Upload widget is only available when the cell has been executed in the
current browser session. Please rerun this cell to enable.

```
Saving IA-B12-26-2-26.csv to IA-B12-26-2-26.csv
Raw dataset: 257 patients, 51 variables
Dataset loaded and renamed: 257 patients, 51 variables
Target column confirmed: B12_Deficiency ✅
```

Out[5]:

|  | Sex | Age | History of Anaemia | Hypertension | Proton Pump Inhibitor | Diabetes Duration | Metformin Duration | Metformin Daily Dose | Metformin Cumulative Dose | Urinary Infection | ... | eGFR | Microalbuminuria | AST | ALT | Anaemia | Hypochromia | Macrocytosis | Leucopenia | Thrombocytopenia | B12\_Deficiency |
| --- | --- | --- | --- | --- | --- | --- | --- | --- | --- | --- | --- | --- | --- | --- | --- | --- | --- | --- | --- | --- | --- |
| 0 | 1 | 50 | 2 | 2 | 2 | 6 | 6 | 1700 | 3723.00 | 10.20 | ... | 108.80 | 10.0 | 32 | 41 | 2 | 2 | 2 | 2 | 2 | 2 |
| 1 | 2 | 61 | 2 | 1 | 1 | 20 | 20 | 1700 | 12410.00 | 34.00 | ... | 67.74 | 8.5 | 23 | 22 | 2 | 2 | 2 | 2 | 2 | 1 |
| 2 | 2 | 65 | 2 | 2 | 2 | 10 | 10 | 1700 | 6205.00 | 17.00 | ... | 76.61 | 13.1 | 14 | 14 | 2 | 2 | 2 | 2 | 2 | 2 |
| 3 | 2 | 60 | 2 | 1 | 1 | 13 | 13 | 1700 | 8066.50 | 22.10 | ... | 90.83 | 20.0 | 15 | 14 | 2 | 2 | 2 | 2 | 2 | 1 |
| 4 | 2 | 65 | 2 | 1 | 1 | 13 | 13 | 2550 | 12099.75 | 33.15 | ... | 59.22 | 0.0 | 17 | 21 | 1 | 1 | 2 | 2 | 2 | 1 |

5 rows × 51 columns

In [6]:

```
# ─────────────────────────────────────────────────────────────
# Replace missing codes 9 → NaN
# (columns with values only in {1, 2, 9})
# ─────────────────────────────────────────────────────────────
required_values = {9, 1, 2}
cols_with_9 = [
    col for col in df.columns
    if set(df[col].dropna().unique()) == required_values
]
df[cols_with_9] = df[cols_with_9].replace(9, np.nan)
print(f"Columns converted (9 → NaN): {cols_with_9}")
```

```
Columns converted (9 → NaN): ['Diabetic Retinopathy', 'Diabetic Nephropathy', 'Erectile Dysfunction', 'Microangiopathy', 'PositionGO', 'Anaemia', 'Hypochromia', 'Macrocytosis']
```

In [7]:

```
# ─────────────────────────────────────────────────────────────
# Detection and replacement of error codes
# (values such as 99, 999 that are statistically aberrant)
# ─────────────────────────────────────────────────────────────
def detect_possible_error_columns(df, error_values=[9, 99, 999, 9999], z_thresh=3):
    suspicious_cols = []
    for col in df.select_dtypes(include=[np.number]).columns:
        col_data = df[col]
        mean, std = col_data.mean(), col_data.std()
        if std == 0 or np.isnan(std):
            continue
        mask = col_data.isin(error_values)
        if mask.any():
            z_scores = np.abs((col_data[mask] - mean) / std)
            if (z_scores > z_thresh).any():
                suspicious_cols.append(col)
    return suspicious_cols

def replace_error_codes_far(df, columns, error_values=[9, 99, 999, 9999], z_thresh=3):
    df = df.copy()
    for col in columns:
        col_data = df[col]
        mean, std = col_data.mean(), col_data.std()
        if std == 0 or np.isnan(std):
            continue
        mask = col_data.isin(error_values)
        if mask.any():
            z_scores = np.abs((col_data[mask] - mean) / std)
            df.loc[mask & (z_scores > z_thresh), col] = np.nan
    return df

# Manually verified columns — English names (post-renaming)
error_columns_verified = [
    'NIS Reflexes Score',
    'NIS Total Score',
    'Haemoglobin',
    'MCV',
    'MCH',
    'MCHC',
    'WBC',
    'Platelets',
    'Microalbuminuria',
    'Leucopenia',
    'Thrombocytopenia',
]
df = replace_error_codes_far(df, error_columns_verified)
print('Missing values after error code correction:')
print(df[error_columns_verified].isna().sum())
```

```
Missing values after error code correction:
NIS Reflexes Score     2
NIS Total Score        2
Haemoglobin            1
MCV                    1
MCH                    1
MCHC                   1
WBC                    1
Platelets              1
Microalbuminuria      10
Leucopenia             1
Thrombocytopenia       1
dtype: int64
```

In [8]:

```
# ─────────────────────────────────────────────────────────────
# Missing values report
# ─────────────────────────────────────────────────────────────
pd.options.display.max_rows = 200
missing_cols    = df.isnull().sum()
missing_percent = (missing_cols / len(df)) * 100
missing_cols    = missing_cols[missing_cols > 0]
missing_percent = missing_percent[missing_cols.index]

missing_data = pd.DataFrame({
    'Missing Values': missing_cols,
    'Percentage (%)': missing_percent.round(1)
})
missing_cols_list = missing_cols.index.tolist()
missing_cols_list += [c for c in error_columns_verified if c not in missing_cols_list]

print(f"Columns with missing values: {len(missing_cols)}\n")
display(missing_data)
```

```
Columns with missing values: 19
```

|  | Missing Values | Percentage (%) |
| --- | --- | --- |
| Diabetic Retinopathy | 8 | 3.1 |
| Diabetic Nephropathy | 10 | 3.9 |
| Erectile Dysfunction | 136 | 52.9 |
| Microangiopathy | 5 | 1.9 |
| PositionGO | 1 | 0.4 |
| NIS Reflexes Score | 2 | 0.8 |
| NIS Total Score | 2 | 0.8 |
| Haemoglobin | 1 | 0.4 |
| MCV | 1 | 0.4 |
| MCH | 1 | 0.4 |
| MCHC | 1 | 0.4 |
| WBC | 1 | 0.4 |
| Platelets | 1 | 0.4 |
| Microalbuminuria | 10 | 3.9 |
| Anaemia | 1 | 0.4 |
| Hypochromia | 1 | 0.4 |
| Macrocytosis | 1 | 0.4 |
| Leucopenia | 1 | 0.4 |
| Thrombocytopenia | 1 | 0.4 |

In [9]:

```
# ─────────────────────────────────────────────────────────────
# Convert binary variables {1, 2} → {1, 0}
# ─────────────────────────────────────────────────────────────
def detect_binary(df):
    return [
        col for col in df.columns
        if len(df[col].dropna().unique()) in [1, 2]
    ]

def convert_binary_12_to_01(df, binary_cols):
    df = df.copy()
    for col in binary_cols:
        df[col] = df[col].replace({1: 1, 2: 0})
    return df, binary_cols

binary_cols = detect_binary(df)
df, converted_columns = convert_binary_12_to_01(df, binary_cols)
print(f"Binary columns converted ({len(converted_columns)}): {converted_columns}")
```

```
Binary columns converted (24): ['Sex', 'History of Anaemia', 'Hypertension', 'Proton Pump Inhibitor', 'Sulfonylureas', 'Insulin', 'Coronary Artery Disease', 'Stroke or TIA', 'Peripheral Artery Disease', 'Macroangiopathy', 'Diabetic Retinopathy', 'Diabetic Nephropathy', 'Erectile Dysfunction', 'Autonomic Neuropathy', 'Peripheral Neuropathy', 'Microangiopathy', 'PositionGO', 'Psychiatric Signs', 'Anaemia', 'Hypochromia', 'Macrocytosis', 'Leucopenia', 'Thrombocytopenia', 'B12_Deficiency']
```

---

## 📊 Section 2 — Exploratory Data Analysis¶

> Target variable distribution, comparative Table 1 (deficient vs. non-deficient).
> Feature selection is performed exclusively by SHAP in Section 5.

In [10]:

```
# ─────────────────────────────────────────────────────────────
# Figure 1 & 2 — Target variable distribution
# ─────────────────────────────────────────────────────────────
TARGET = 'B12_Deficiency'
counts  = df[TARGET].value_counts()
majority = counts.get(0, 0)
minority = counts.get(1, 0)

fig, axes = plt.subplots(1, 2, figsize=(12, 5))

# Bar chart
sns.countplot(x=TARGET, data=df,
              palette=['#2ECC71', '#E74C3C'], ax=axes[0])
axes[0].set_title('Distribution — B12 Deficiency Status')
axes[0].set_xlabel('Deficiency Status (1 = Deficient, 0 = Non-Deficient)')
axes[0].set_ylabel('Number of Patients')
for p in axes[0].patches:
    axes[0].annotate(f'{int(p.get_height())}',
                     (p.get_x() + p.get_width()/2, p.get_height() + 1),
                     ha='center', fontsize=11)

# Pie chart
axes[1].pie(
    [majority, minority],
    labels=['Non-Deficient', 'Deficient'],
    autopct='%1.1f%%', startangle=90,
    colors=['#2ECC71', '#E74C3C']
)
axes[1].set_title('Proportion — B12 Deficiency Status')

plt.tight_layout()
plt.savefig(os.path.join(FIGURES_PATH, 'Fig1_B12_deficiency_distribution.png'), **SAVEFIG_KW)
plt.show()

print(f"Non-Deficient: {majority} patients ({majority/len(df)*100:.1f}%)")
print(f"Deficient: {minority} patients ({minority/len(df)*100:.1f}%)")
```

```
Non-Deficient: 162 patients (63.0%)
Deficient: 95 patients (37.0%)
```

In [11]:

```
# ─────────────────────────────────────────────────────────────
# Table 1 — Comparative statistics by B12 status
# Mann-Whitney / t-test for continuous, Chi2 / Fisher for categorical
# ─────────────────────────────────────────────────────────────
def table1(df, target_col='B12_Deficiency', cat_threshold=5):
    table = []
    for col in df.columns:
        if col == target_col:
            continue
        group_def    = df[df[target_col] == 1][col].dropna()
        group_no_def = df[df[target_col] == 0][col].dropna()
        n_unique = df[col].nunique()

        if n_unique <= cat_threshold:
            contingency = pd.crosstab(df[col], df[target_col])
            if contingency.shape == (2, 2):
                if (contingency.values < 5).any():
                    _, p_value = stats.fisher_exact(contingency)
                else:
                    chi2, p_value, _, _ = stats.chi2_contingency(contingency)
            else:
                chi2, p_value, _, _ = stats.chi2_contingency(contingency)

            counts_no_def = df[df[target_col]==0][col].value_counts(normalize=True).sort_index()
            counts_def    = df[df[target_col]==1][col].value_counts(normalize=True).sort_index()

            val_no_def = ', '.join([
                f"{k}: {df[df[target_col]==0][col].value_counts()[k]} ({counts_no_def[k]*100:.1f}%)"
                for k in counts_no_def.index
            ])
            val_def = ', '.join([
                f"{k}: {df[df[target_col]==1][col].value_counts()[k]} ({counts_def[k]*100:.1f}%)"
                for k in counts_def.index
            ])
            table.append([col, val_no_def, val_def, p_value])
        else:
            p_norm_def    = stats.shapiro(group_def)[1]    if len(group_def)    < 5000 else 0
            p_norm_no_def = stats.shapiro(group_no_def)[1] if len(group_no_def) < 5000 else 0
            if p_norm_def > 0.05 and p_norm_no_def > 0.05:
                _, p_value   = stats.ttest_ind(group_def, group_no_def)
                val_def    = f"{group_def.mean():.2f} \u00b1 {group_def.std():.2f}"
                val_no_def = f"{group_no_def.mean():.2f} \u00b1 {group_no_def.std():.2f}"
            else:
                _, p_value   = stats.mannwhitneyu(group_def, group_no_def)
                val_def    = f"{group_def.median():.2f} ({group_def.quantile(0.25):.2f}\u2013{group_def.quantile(0.75):.2f})"
                val_no_def = f"{group_no_def.median():.2f} ({group_no_def.quantile(0.25):.2f}\u2013{group_no_def.quantile(0.75):.2f})"
            table.append([col, val_no_def, val_def, p_value])

    return pd.DataFrame(table, columns=['Variable', 'Non-Deficient', 'Deficient', 'p-value'])

table1_df = table1(df, TARGET)
table1_csv = os.path.join(TABLES_PATH, 'Table1_comparative_stats.csv')
table1_df.to_csv(table1_csv, index=False)
print(f"Table 1 saved → {table1_csv}")
display(table1_df)
```

```
Table 1 saved → outputs/tables/Table1_comparative_stats.csv
```

|  | Variable | Non-Deficient | Deficient | p-value |
| --- | --- | --- | --- | --- |
| 0 | Sex | 0: 97 (59.9%), 1: 65 (40.1%) | 0: 39 (41.1%), 1: 56 (58.9%) | 0.005289 |
| 1 | Age | 61.00 (54.25–65.00) | 60.00 (56.00–65.00) | 0.740920 |
| 2 | History of Anaemia | 0: 157 (96.9%), 1: 5 (3.1%) | 0: 90 (94.7%), 1: 5 (5.3%) | 0.591315 |
| 3 | Hypertension | 0: 66 (40.7%), 1: 96 (59.3%) | 0: 34 (35.8%), 1: 61 (64.2%) | 0.513532 |
| 4 | Proton Pump Inhibitor | 0: 103 (63.6%), 1: 59 (36.4%) | 0: 64 (67.4%), 1: 31 (32.6%) | 0.631887 |
| 5 | Diabetes Duration | 9.00 (6.00–14.50) | 10.00 (7.00–15.00) | 0.383449 |
| 6 | Metformin Duration | 9.00 (6.00–13.00) | 10.00 (6.50–14.50) | 0.410156 |
| 7 | Metformin Daily Dose | 850: 15 (9.3%), 1700: 59 (36.4%), 2000: 6 (3.7... | 850: 5 (5.3%), 1700: 42 (44.2%), 2000: 1 (1.1%... | 0.300706 |
| 8 | Metformin Cumulative Dose | 6205.00 (3723.00–10238.25) | 7446.00 (4343.50–10858.75) | 0.341114 |
| 9 | Urinary Infection | 17.00 (10.20–28.05) | 20.40 (11.90–29.75) | 0.341114 |
| 10 | Girerd Score | 4.00 (2.00–5.00) | 4.00 (3.00–5.00) | 0.360492 |
| 11 | Treatment Adherence | 1: 10 (6.2%), 2: 78 (48.1%), 3: 74 (45.7%) | 1: 7 (7.4%), 2: 45 (47.4%), 3: 43 (45.3%) | 0.932727 |
| 12 | Sulfonylureas | 0: 84 (51.9%), 1: 78 (48.1%) | 0: 43 (45.3%), 1: 52 (54.7%) | 0.373166 |
| 13 | Insulin | 0: 100 (61.7%), 1: 62 (38.3%) | 0: 67 (70.5%), 1: 28 (29.5%) | 0.196441 |
| 14 | Coronary Artery Disease | 0: 134 (82.7%), 1: 28 (17.3%) | 0: 77 (81.1%), 1: 18 (18.9%) | 0.867182 |
| 15 | Stroke or TIA | 0: 154 (95.1%), 1: 8 (4.9%) | 0: 90 (94.7%), 1: 5 (5.3%) | 1.000000 |
| 16 | Peripheral Artery Disease | 0: 160 (98.8%), 1: 2 (1.2%) | 0: 92 (96.8%), 1: 3 (3.2%) | 0.362006 |
| 17 | Macroangiopathy | 0: 130 (80.2%), 1: 32 (19.8%) | 0: 71 (74.7%), 1: 24 (25.3%) | 0.380831 |
| 18 | Diabetic Retinopathy | 0.0: 118 (75.2%), 1.0: 39 (24.8%) | 0.0: 72 (78.3%), 1.0: 20 (21.7%) | 0.688296 |
| 19 | Diabetic Nephropathy | 0.0: 114 (74.0%), 1.0: 40 (26.0%) | 0.0: 56 (60.2%), 1.0: 37 (39.8%) | 0.033283 |
| 20 | Erectile Dysfunction | 0.0: 34 (52.3%), 1.0: 31 (47.7%) | 0.0: 25 (44.6%), 1.0: 31 (55.4%) | 0.510104 |
| 21 | Autonomic Neuropathy | 0: 126 (77.8%), 1: 36 (22.2%) | 0: 54 (56.8%), 1: 41 (43.2%) | 0.000685 |
| 22 | Peripheral Neuropathy | 0: 67 (41.4%), 1: 95 (58.6%) | 0: 50 (52.6%), 1: 45 (47.4%) | 0.104787 |
| 23 | Microangiopathy | 0.0: 37 (23.3%), 1.0: 122 (76.7%) | 0.0: 15 (16.1%), 1.0: 78 (83.9%) | 0.233853 |
| 24 | BMI | 30.73 (27.85–33.69) | 29.62 (26.75–31.69) | 0.020770 |
| 25 | Systolic BP | 140.00 (120.00–150.00) | 140.00 (130.00–150.00) | 0.283636 |
| 26 | Diastolic BP | 70.00 (70.00–80.00) | 70.00 (70.00–80.00) | 0.533805 |
| 27 | PositionGO | 0.0: 7 (4.3%), 1.0: 154 (95.7%) | 0.0: 6 (6.3%), 1.0: 89 (93.7%) | 0.690472 |
| 28 | Psychiatric Signs | 0: 145 (89.5%), 1: 17 (10.5%) | 0: 87 (91.6%), 1: 8 (8.4%) | 0.746512 |
| 29 | DN4 Score | 1.50 (0.00–4.00) | 2.00 (0.00–4.00) | 0.353590 |
| 30 | NIS Reflexes Score | 1.00 (0.00–4.00) | 0.00 (0.00–3.00) | 0.181067 |
| 31 | NIS Sensitivity Score | 2.00 (0.00–4.00) | 2.00 (0.00–4.00) | 0.989187 |
| 32 | NIS Total Score | 4.00 (1.00–7.00) | 3.00 (0.50–6.00) | 0.390863 |
| 33 | Haemoglobin | 13.20 (12.45–14.00) | 13.20 (12.20–14.38) | 0.879563 |
| 34 | MCV | 85.20 (81.62–87.78) | 86.20 (82.35–89.17) | 0.172792 |
| 35 | MCH | 28.85 (27.30–29.90) | 29.30 (27.30–30.40) | 0.276382 |
| 36 | MCHC | 33.70 (32.90–34.50) | 33.55 (32.62–34.60) | 0.804920 |
| 37 | WBC | 6965.00 (5685.00–8345.75) | 7125.00 (5880.00–8345.00) | 0.576438 |
| 38 | Platelets | 251.00 (214.25–289.75) | 251.00 (220.50–294.75) | 0.555688 |
| 39 | Fasting Glucose | 1.93 (1.56–2.94) | 1.75 (1.42–2.33) | 0.012473 |
| 40 | HbA1c | 8.75 (7.62–10.10) | 7.90 (6.70–8.95) | 0.000014 |
| 41 | eGFR | 89.65 (78.06–92.80) | 88.02 (72.78–94.10) | 0.199178 |
| 42 | Microalbuminuria | 10.98 (5.03–30.38) | 21.00 (6.70–60.80) | 0.008899 |
| 43 | AST | 16.00 (14.00–20.00) | 16.00 (14.00–19.00) | 0.353643 |
| 44 | ALT | 17.00 (14.00–26.00) | 17.00 (13.50–21.00) | 0.112295 |
| 45 | Anaemia | 0.0: 130 (80.2%), 1.0: 32 (19.8%) | 0.0: 69 (73.4%), 1.0: 25 (26.6%) | 0.265834 |
| 46 | Hypochromia | 0.0: 113 (69.8%), 1.0: 49 (30.2%) | 0.0: 69 (73.4%), 1.0: 25 (26.6%) | 0.632523 |
| 47 | Macrocytosis | 0.0: 161 (99.4%), 1.0: 1 (0.6%) | 0.0: 94 (100.0%) | 1.000000 |
| 48 | Leucopenia | 0.0: 162 (100.0%) | 0.0: 94 (100.0%) | 1.000000 |
| 49 | Thrombocytopenia | 0.0: 162 (100.0%) | 0.0: 94 (100.0%) | 1.000000 |

---

## 🔧 Section 3 — Preprocessing¶

> Stratified train/test split (80/20), column type detection, distribution detection, type-aware imputation.

In [12]:

```
# ─────────────────────────────────────────────────────────────
# Stratified Train / Test split
# ─────────────────────────────────────────────────────────────
X = df.drop(columns=[TARGET])
y = df[TARGET]

X_train, X_test, y_train, y_test = train_test_split(
    X, y,
    test_size=0.20,
    stratify=y,
    random_state=SEED
)

print(f"Train: {X_train.shape[0]} patients  |  Test: {X_test.shape[0]} patients")
print(f"Train prevalence: {y_train.mean():.1%}  |  Test prevalence: {y_test.mean():.1%}")
```

```
Train: 205 patients  |  Test: 52 patients
Train prevalence: 37.1%  |  Test prevalence: 36.5%
```

In [13]:

```
# ─────────────────────────────────────────────────────────────
# Column type detection
# Binary / Ordinal / Continuous
# ─────────────────────────────────────────────────────────────
def detect_column_types(df, ordinal_threshold=10):
    binary_cols, ordinal_cols, continuous_cols = [], [], []
    for col in df.columns:
        unique_vals = df[col].dropna().unique()
        n_unique = len(unique_vals)
        if n_unique in [1, 2]:
            binary_cols.append(col)
        elif (
            pd.api.types.is_numeric_dtype(df[col])
            and n_unique <= ordinal_threshold
            and np.all(unique_vals == unique_vals.astype(int))
        ):
            ordinal_cols.append(col)
        else:
            continuous_cols.append(col)
    return binary_cols, ordinal_cols, continuous_cols

def detect_distribution_columns(df, skew_threshold=1.0):
    normal_cols, skewed_cols = [], []
    for col in df.select_dtypes(include=['number']).columns:
        skewness = df[col].dropna().skew()
        if abs(skewness) < skew_threshold:
            normal_cols.append(col)
        else:
            skewed_cols.append(col)
    return normal_cols, skewed_cols

binary_cols, ordinal_cols, continuous_cols = detect_column_types(X_train)
normal_cols,  skewed_cols                  = detect_distribution_columns(X_train)

print(f"Binary    : {len(binary_cols)}")
print(f"Ordinal   : {len(ordinal_cols)}")
print(f"Continuous: {len(continuous_cols)} ({len(skewed_cols)} skewed)")
```

```
Binary    : 23
Ordinal   : 6
Continuous: 21 (21 skewed)
```

In [14]:

```
# ─────────────────────────────────────────────────────────────
# Type-aware imputation
#   Binary     → mode (most_frequent)
#   Ordinal    → median
#   Continuous → mean (symmetric) or median (skewed)
# ─────────────────────────────────────────────────────────────
def build_type_aware_imputer(binary_cols, ordinal_cols, continuous_cols, skewed_cols=[]):
    transformers = []
    if binary_cols:
        transformers.append(('binary_imputer', SimpleImputer(strategy='most_frequent'), binary_cols))
    if ordinal_cols:
        transformers.append(('ordinal_imputer', SimpleImputer(strategy='median'), ordinal_cols))
    skewed_continuous = [c for c in continuous_cols if c in skewed_cols]
    mean_continuous   = [c for c in continuous_cols if c not in skewed_cols]
    if mean_continuous:
        transformers.append(('cont_mean_imputer', SimpleImputer(strategy='mean'), mean_continuous))
    if skewed_continuous:
        transformers.append(('cont_median_imputer', SimpleImputer(strategy='median'), skewed_continuous))
    return ColumnTransformer(transformers=transformers, remainder='passthrough')

imputer = build_type_aware_imputer(binary_cols, ordinal_cols, continuous_cols, skewed_cols)

X_train_imputed = imputer.fit_transform(X_train)
X_test_imputed  = imputer.transform(X_test)

# Restore column names
feature_names = imputer.get_feature_names_out()
X_train_imputed = pd.DataFrame(X_train_imputed, columns=feature_names, index=X_train.index)
X_test_imputed  = pd.DataFrame(X_test_imputed,  columns=feature_names, index=X_test.index)
X_train_imputed.columns = [c.split('__')[-1] for c in X_train_imputed.columns]
X_test_imputed.columns  = [c.split('__')[-1] for c in X_test_imputed.columns]
X_train_imputed = X_train_imputed[X_train.columns]
X_test_imputed  = X_test_imputed[X_test.columns]

print("Imputation complete — remaining missing values:")
print(X_train_imputed.isna().sum().sum())
```

```
Imputation complete — remaining missing values:
0
```

---

## ⚖️ Section 4 — Resampling Strategy Selection¶

> Comparison of five class imbalance handling strategies using 5-fold cross-validation:
> No Resampling / BorderlineSMOTE / ADASYN / scale\_pos\_weight=1.70 / scale\_pos\_weight=4.0.
> Selection criterion: MCC (robust to class imbalance). Final strategy selected: scale\_pos\_weight=4.0.

In [15]:

```
# ─────────────────────────────────────────────────────────────
# Resampling strategy comparison
# ─────────────────────────────────────────────────────────────
resampling_strategies = {
    'No Resampling'   : None,
    'BorderlineSMOTE' : BorderlineSMOTE(random_state=SEED),
    'ADASYN'          : ADASYN(random_state=SEED)
}

cv = StratifiedKFold(n_splits=5, shuffle=True, random_state=SEED)
results = []

for name, sampler in resampling_strategies.items():
    print(f"  Strategy: {name}")
    X_res, y_res = X_train_imputed.copy(), y_train.copy()
    if sampler is not None:
        X_res, y_res = sampler.fit_resample(X_res, y_res)

    model  = xgb.XGBClassifier(random_state=SEED, eval_metric='logloss', n_jobs=-1)
    y_pred = cross_val_predict(model, X_res, y_res, cv=cv)

    tn, fp, fn, tp = confusion_matrix(y_res, y_pred).ravel()
    results.append({
        'Strategy'         : name,
        'MCC'              : matthews_corrcoef(y_res, y_pred),
        'Balanced Accuracy': balanced_accuracy_score(y_res, y_pred),
        'Sensitivity'      : tp / (tp + fn),
        'Specificity'      : tn / (tn + fp),
    })

results_resampling = pd.DataFrame(results).sort_values('MCC', ascending=False)
results_resampling.to_csv(os.path.join(TABLES_PATH, 'Table_resampling_comparison.csv'), index=False)
print("\nResampling strategy comparison results:")
display(results_resampling)

# Figure
metrics_to_plot = ['MCC', 'Balanced Accuracy', 'Sensitivity', 'Specificity']
fig, axes = plt.subplots(1, 4, figsize=(16, 5))
for ax, metric in zip(axes, metrics_to_plot):
    sns.barplot(x='Strategy', y=metric, data=results_resampling, palette='Set1', ax=ax)
    ax.set_title(metric)
    ax.set_ylim(0, 1)
    ax.set_xticklabels(ax.get_xticklabels(), rotation=40, ha='right')
    for idx, val in enumerate(results_resampling[metric]):
        ax.text(idx, val + 0.02, f'{val:.2f}', ha='center', fontsize=9)
plt.suptitle('Resampling Strategy Comparison (5-Fold CV — XGBoost Baseline)', fontsize=13, fontweight='bold')
plt.tight_layout()
plt.savefig(os.path.join(FIGURES_PATH, 'Fig2_resampling_comparison.png'), **SAVEFIG_KW)
plt.show()
```

```
  Strategy: No Resampling
  Strategy: BorderlineSMOTE
  Strategy: ADASYN

Resampling strategy comparison results:
```

|  | Strategy | MCC | Balanced Accuracy | Sensitivity | Specificity |
| --- | --- | --- | --- | --- | --- |
| 1 | BorderlineSMOTE | 0.403696 | 0.701550 | 0.728682 | 0.674419 |
| 2 | ADASYN | 0.401610 | 0.699682 | 0.748201 | 0.651163 |
| 0 | No Resampling | 0.025137 | 0.511781 | 0.302632 | 0.720930 |

In [16]:

```
# ─────────────────────────────────────────────────────────────
# Imbalance handling strategy comparison
# 4 strategies: No Resampling, BorderlineSMOTE, ADASYN,
#               scale_pos_weight (native XGBoost)
# All evaluated on X_train_imputed (all features, baseline XGBoost)
# ─────────────────────────────────────────────────────────────

cv = StratifiedKFold(n_splits=5, shuffle=True, random_state=SEED)
results = []

# ── Strategies 1–3 : resampling-based ──────────────────────
resampling_strategies = {
    'No Resampling'   : None,
    'BorderlineSMOTE' : BorderlineSMOTE(random_state=SEED),
    'ADASYN'          : ADASYN(random_state=SEED),
}

for name, sampler in resampling_strategies.items():
    print(f"  Strategy: {name}")
    X_res, y_res = X_train_imputed.copy(), y_train.copy()
    if sampler is not None:
        X_res, y_res = sampler.fit_resample(X_res, y_res)

    model  = xgb.XGBClassifier(random_state=SEED, eval_metric='logloss', n_jobs=-1)
    y_pred = cross_val_predict(model, X_res, y_res, cv=cv)

    tn, fp, fn, tp = confusion_matrix(y_res, y_pred).ravel()
    results.append({
        'Strategy'         : name,
        'MCC'              : matthews_corrcoef(y_res, y_pred),
        'Balanced Accuracy': balanced_accuracy_score(y_res, y_pred),
        'Sensitivity'      : tp / (tp + fn),
        'Specificity'      : tn / (tn + fp),
    })

# ── Strategy 4 : scale_pos_weight (native XGBoost) ─────────
# ratio non-deficient / deficient = 162 / 95 ≈ 1.71
# but we use the value selected by BayesSearch (4.0)
# to keep this comparison representative of the final pipeline
print(f"  Strategy: scale_pos_weight=4.0")
neg, pos = (y_train == 0).sum(), (y_train == 1).sum()
spw = neg / pos  # natural ratio ≈ 1.71 — shown here as reference
print(f"    (natural ratio neg/pos = {spw:.2f} | BayesSearch-optimised value = 4.0)")

for spw_val, label in [(neg/pos, f'scale_pos_weight={neg/pos:.2f} (natural)'),
                        (4.0,    'scale_pos_weight=4.0 (optimised)')]:
    model_spw = xgb.XGBClassifier(
        random_state=SEED, eval_metric='logloss', n_jobs=-1,
        scale_pos_weight=spw_val
    )
    y_pred_spw = cross_val_predict(model_spw, X_train_imputed, y_train, cv=cv)
    tn, fp, fn, tp = confusion_matrix(y_train, y_pred_spw).ravel()
    results.append({
        'Strategy'         : label,
        'MCC'              : matthews_corrcoef(y_train, y_pred_spw),
        'Balanced Accuracy': balanced_accuracy_score(y_train, y_pred_spw),
        'Sensitivity'      : tp / (tp + fn),
        'Specificity'      : tn / (tn + fp),
    })

# ── Results ─────────────────────────────────────────────────
results_resampling = pd.DataFrame(results).sort_values('MCC', ascending=False)
results_resampling.to_csv(os.path.join(TABLES_PATH, 'Table_resampling_comparison.csv'), index=False)
print("\nImbalance handling strategy comparison results:")
display(results_resampling)

# Figure
metrics_to_plot = ['MCC', 'Balanced Accuracy', 'Sensitivity', 'Specificity']
fig, axes = plt.subplots(1, 4, figsize=(20, 5))
for ax, metric in zip(axes, metrics_to_plot):
    sns.barplot(x='Strategy', y=metric, data=results_resampling, palette='Set1', ax=ax)
    ax.set_title(metric)
    ax.set_ylim(0, 1)
    ax.set_xticklabels(ax.get_xticklabels(), rotation=40, ha='right')
    for idx, val in enumerate(results_resampling[metric]):
        ax.text(idx, val + 0.02, f'{val:.3f}', ha='center', fontsize=8)
plt.suptitle('Imbalance Handling Strategy Comparison (5-Fold CV — XGBoost Baseline)',
             fontsize=13, fontweight='bold')
plt.tight_layout()
plt.savefig(os.path.join(FIGURES_PATH, 'Fig2_resampling_comparison.png'), **SAVEFIG_KW)
plt.show()
```

```
  Strategy: No Resampling
  Strategy: BorderlineSMOTE
  Strategy: ADASYN
  Strategy: scale_pos_weight=4.0
    (natural ratio neg/pos = 1.70 | BayesSearch-optimised value = 4.0)

Imbalance handling strategy comparison results:
```

|  | Strategy | MCC | Balanced Accuracy | Sensitivity | Specificity |
| --- | --- | --- | --- | --- | --- |
| 1 | BorderlineSMOTE | 0.403696 | 0.701550 | 0.728682 | 0.674419 |
| 2 | ADASYN | 0.401610 | 0.699682 | 0.748201 | 0.651163 |
| 4 | scale\_pos\_weight=4.0 (optimised) | 0.130089 | 0.566656 | 0.513158 | 0.620155 |
| 3 | scale\_pos\_weight=1.70 (natural) | 0.100877 | 0.550439 | 0.434211 | 0.666667 |
| 0 | No Resampling | 0.025137 | 0.511781 | 0.302632 | 0.720930 |

---

## 🤖 Section 5 — Model Selection & SHAP Feature Selection¶

> **Step 5.1**: Baseline XGBoost trained on all features to compute SHAP importances.

> **Step 5.2**: Selection of features with non-zero SHAP importance.

> **Step 5.3**: Comparison of 4 models (XGBoost, Random Forest, SVM, Logistic Regression L2)
> on selected features — 5-fold cross-validation without resampling
> (class imbalance handled via scale\_pos\_weight within XGBoost only).

> *Note: SHAP-based selection is consistent with the non-linear architecture of XGBoost.
> A Lasso (linear) approach is not appropriate here.*

In [17]:

```
# ─────────────────────────────────────────────────────────────
# 5.1 — Baseline XGBoost (all features) for SHAP
# ─────────────────────────────────────────────────────────────
xgb_baseline = xgb.XGBClassifier(random_state=SEED, eval_metric='logloss')
xgb_baseline.fit(X_train_imputed, y_train)

y_pred_base = xgb_baseline.predict(X_test_imputed)
y_prob_base = xgb_baseline.predict_proba(X_test_imputed)[:, 1]

tn, fp, fn, tp = confusion_matrix(y_test, y_pred_base).ravel()
print("Baseline XGBoost (test set)")
print(f"  ROC-AUC    : {roc_auc_score(y_test, y_prob_base):.3f}")
print(f"  MCC        : {matthews_corrcoef(y_test, y_pred_base):.3f}")
print(f"  Sensitivity: {tp/(tp+fn):.3f}  |  Specificity: {tn/(tn+fp):.3f}")
```

```
Baseline XGBoost (test set)
  ROC-AUC    : 0.649
  MCC        : 0.153
  Sensitivity: 0.316  |  Specificity: 0.818
```

In [18]:

```
# ─────────────────────────────────────────────────────────────
# 5.2 — SHAP Feature Importance (baseline)
# Select features with SHAP importance > 0
# ─────────────────────────────────────────────────────────────
explainer_base  = shap.Explainer(xgb_baseline, X_train_imputed)
shap_values_base = explainer_base(X_train_imputed)

# Figure SHAP baseline
shap.summary_plot(shap_values_base, X_train_imputed, plot_type='bar', show=False)
plt.title('SHAP Feature Importance — XGBoost Baseline (all features)', fontsize=12)
plt.savefig(os.path.join(FIGURES_PATH, 'Fig3_SHAP_baseline_importance.png'), **SAVEFIG_KW)
plt.show()

# Sélection
shap_importance = pd.DataFrame({
    'feature'       : X_train_imputed.columns,
    'mean_abs_shap' : np.abs(shap_values_base.values).mean(axis=0)
})
important_features = shap_importance.loc[
    shap_importance['mean_abs_shap'] > 0, 'feature'
].tolist()

print(f"Retained features (SHAP > 0): {len(important_features)}")
print(shap_importance.sort_values('mean_abs_shap', ascending=False).head(20))

# Sous-ensembles
X_train_selected = X_train_imputed[important_features]
X_test_selected  = X_test_imputed[important_features]
```

```
Retained features (SHAP > 0): 36
                      feature  mean_abs_shap
40                      HbA1c       1.052218
42           Microalbuminuria       0.873135
29                  DN4 Score       0.663453
21       Autonomic Neuropathy       0.654795
24                        BMI       0.572482
41                       eGFR       0.542740
34                        MCV       0.393261
39            Fasting Glucose       0.325983
37                        WBC       0.321662
38                  Platelets       0.280485
36                       MCHC       0.256441
44                        ALT       0.235360
33                Haemoglobin       0.208561
1                         Age       0.185438
26               Diastolic BP       0.162861
30         NIS Reflexes Score       0.135970
8   Metformin Cumulative Dose       0.135658
35                        MCH       0.134202
25                Systolic BP       0.132118
13                    Insulin       0.125783
```

In [19]:

```
# ─────────────────────────────────────────────────────────────
# 5.3 — 4-model comparison (5-fold CV, no resampling)
#   XGBoost / Random Forest / SVM / Logistic Regression (L2)
# ─────────────────────────────────────────────────────────────
models = {
    'XGBoost'                  : xgb.XGBClassifier(random_state=SEED, eval_metric='logloss', n_jobs=-1),
    'Random Forest'            : RandomForestClassifier(n_estimators=100, random_state=SEED, n_jobs=-1),
    'SVM'                      : SVC(kernel='rbf', probability=True, class_weight='balanced', random_state=SEED),
    'Logistic Regression (L2)' : LogisticRegression(penalty='l2', solver='liblinear', random_state=SEED),
}

cv5 = StratifiedKFold(n_splits=5, shuffle=True, random_state=SEED)
results_models = []

for name, model in models.items():
    print(f"  Running {name}...")
    y_pred  = cross_val_predict(model, X_train_selected, y_train, cv=cv5)
    y_proba = cross_val_predict(model, X_train_selected, y_train, cv=cv5, method='predict_proba')[:, 1]

    tn, fp, fn, tp = confusion_matrix(y_train, y_pred).ravel()
    results_models.append({
        'Model'            : name,
        'ROC-AUC'          : roc_auc_score(y_train, y_proba),
        'MCC'              : matthews_corrcoef(y_train, y_pred),
        'Balanced Accuracy': balanced_accuracy_score(y_train, y_pred),
        'Sensitivity'      : tp / (tp + fn),
        'Specificity'      : tn / (tn + fp),
    })

results_models_df = pd.DataFrame(results_models).sort_values('MCC', ascending=False)
results_models_df.to_csv(os.path.join(TABLES_PATH, 'Table_model_comparison.csv'), index=False)
print("\nModel comparison (train CV):")
display(results_models_df)

# Figure
metrics_to_plot = ['ROC-AUC', 'MCC', 'Sensitivity', 'Specificity']
fig, axes = plt.subplots(1, 4, figsize=(18, 5))
for ax, metric in zip(axes, metrics_to_plot):
    sns.barplot(x='Model', y=metric, data=results_models_df, palette='Set2', ax=ax)
    ax.set_title(metric)
    ax.set_ylim(0, 1)
    ax.set_xticklabels(ax.get_xticklabels(), rotation=40, ha='right')
    for idx, val in enumerate(results_models_df[metric]):
        ax.text(idx, val + 0.02, f'{val:.2f}', ha='center', fontsize=9)
plt.suptitle('Model Comparison (5-Fold CV, no resampling — SHAP-selected features)', fontsize=13, fontweight='bold')
plt.tight_layout()
plt.savefig(os.path.join(FIGURES_PATH, 'Fig4_model_comparison.png'), **SAVEFIG_KW)
plt.show()
```

```
  Running XGBoost...
  Running Random Forest...
  Running SVM...
  Running Logistic Regression (L2)...

Model comparison (train CV):
```

|  | Model | ROC-AUC | MCC | Balanced Accuracy | Sensitivity | Specificity |
| --- | --- | --- | --- | --- | --- | --- |
| 3 | Logistic Regression (L2) | 0.685027 | 0.264236 | 0.631375 | 0.526316 | 0.736434 |
| 0 | XGBoost | 0.610261 | 0.076341 | 0.536924 | 0.368421 | 0.705426 |
| 1 | Random Forest | 0.590779 | 0.048527 | 0.518462 | 0.184211 | 0.852713 |
| 2 | SVM | 0.595471 | -0.123086 | 0.438137 | 0.302632 | 0.573643 |

---

## 🎯 Section 6 — Hyperparameter Optimization & Evaluation¶

> Bayesian optimisation (BayesSearchCV, 100 iterations, 5-fold CV, scoring = MCC).

> Final evaluation: CV metrics (mean ± SD), test set, calibration curve, bootstrap 95% CI.

In [20]:

```
# ─────────────────────────────────────────────────────────────
# Bayesian search space
# ─────────────────────────────────────────────────────────────
#param_space_xgb = {
#    'xgb__n_estimators'     : Integer(100, 300),
#    'xgb__learning_rate'    : Real(0.01, 0.3, prior='log-uniform'),
#    'xgb__max_depth'        : Integer(3, 8),
#    'xgb__min_child_weight' : Integer(1, 8),
#    'xgb__gamma'            : Real(0, 3),
#    'xgb__subsample'        : Real(0.5, 1.0),
#    'xgb__colsample_bytree' : Real(0.5, 1.0),
#    'xgb__reg_alpha'        : Real(0, 10),
#    'xgb__reg_lambda'       : Real(0, 10),
#    'xgb__scale_pos_weight' : Real(1, 4),
#}
param_space_xgb = {
    'xgb__n_estimators'     : Integer(100, 500),
    'xgb__learning_rate'    : Real(0.01, 0.2,  prior='log-uniform'),
    'xgb__max_depth'        : Integer(2, 5),       # ← reduced (shallower trees)
    'xgb__min_child_weight' : Integer(3, 10),      # ← increased (more conservative)
    'xgb__gamma'            : Real(0.5, 5),        # ← minimum raised (forced pruning)
    'xgb__subsample'        : Real(0.5, 0.9),      # ← max lowered (more subsampling)
    'xgb__colsample_bytree' : Real(0.5, 0.9),      # ← max lowered
    'xgb__reg_alpha'        : Real(1, 20),         # ← minimum raised (stronger L1)
    'xgb__reg_lambda'       : Real(5, 30),         # ← raised (stronger L2)
    'xgb__scale_pos_weight' : Real(1, 4),
}

cv_stratified = StratifiedKFold(n_splits=5, shuffle=True, random_state=SEED)

xgb_pipeline = ImbPipeline([
    ('xgb', xgb.XGBClassifier(random_state=SEED, eval_metric='logloss', n_jobs=-1))
])

bayes_search = BayesSearchCV(
    estimator=xgb_pipeline,
    search_spaces=param_space_xgb,
    n_iter=100,
    cv=cv_stratified,
    scoring=make_scorer(matthews_corrcoef),
    n_jobs=-1,
    random_state=SEED,
    verbose=1
)

print("Running BayesSearchCV (100 iterations × 5 folds)...")
start = time.time()
bayes_search.fit(X_train_selected, y_train)
print(f"Completed in {time.time()-start:.0f}s")

best_model = bayes_search.best_estimator_
xgb_model  = best_model.named_steps['xgb']

print("\nBest parameters:")
print(bayes_search.best_params_)
```

```
Running BayesSearchCV (100 iterations × 5 folds)...
Fitting 5 folds for each of 1 candidates, totalling 5 fits
Fitting 5 folds for each of 1 candidates, totalling 5 fits
Fitting 5 folds for each of 1 candidates, totalling 5 fits
Fitting 5 folds for each of 1 candidates, totalling 5 fits
Fitting 5 folds for each of 1 candidates, totalling 5 fits
Fitting 5 folds for each of 1 candidates, totalling 5 fits
Fitting 5 folds for each of 1 candidates, totalling 5 fits
Fitting 5 folds for each of 1 candidates, totalling 5 fits
Fitting 5 folds for each of 1 candidates, totalling 5 fits
Fitting 5 folds for each of 1 candidates, totalling 5 fits
Fitting 5 folds for each of 1 candidates, totalling 5 fits
Fitting 5 folds for each of 1 candidates, totalling 5 fits
Fitting 5 folds for each of 1 candidates, totalling 5 fits
Fitting 5 folds for each of 1 candidates, totalling 5 fits
Fitting 5 folds for each of 1 candidates, totalling 5 fits
Fitting 5 folds for each of 1 candidates, totalling 5 fits
Fitting 5 folds for each of 1 candidates, totalling 5 fits
Fitting 5 folds for each of 1 candidates, totalling 5 fits
Fitting 5 folds for each of 1 candidates, totalling 5 fits
Fitting 5 folds for each of 1 candidates, totalling 5 fits
Fitting 5 folds for each of 1 candidates, totalling 5 fits
Fitting 5 folds for each of 1 candidates, totalling 5 fits
Fitting 5 folds for each of 1 candidates, totalling 5 fits
Fitting 5 folds for each of 1 candidates, totalling 5 fits
Fitting 5 folds for each of 1 candidates, totalling 5 fits
Fitting 5 folds for each of 1 candidates, totalling 5 fits
Fitting 5 folds for each of 1 candidates, totalling 5 fits
Fitting 5 folds for each of 1 candidates, totalling 5 fits
Fitting 5 folds for each of 1 candidates, totalling 5 fits
Fitting 5 folds for each of 1 candidates, totalling 5 fits
Fitting 5 folds for each of 1 candidates, totalling 5 fits
Fitting 5 folds for each of 1 candidates, totalling 5 fits
Fitting 5 folds for each of 1 candidates, totalling 5 fits
Fitting 5 folds for each of 1 candidates, totalling 5 fits
Fitting 5 folds for each of 1 candidates, totalling 5 fits
Fitting 5 folds for each of 1 candidates, totalling 5 fits
Fitting 5 folds for each of 1 candidates, totalling 5 fits
Fitting 5 folds for each of 1 candidates, totalling 5 fits
Fitting 5 folds for each of 1 candidates, totalling 5 fits
Fitting 5 folds for each of 1 candidates, totalling 5 fits
Fitting 5 folds for each of 1 candidates, totalling 5 fits
Fitting 5 folds for each of 1 candidates, totalling 5 fits
Fitting 5 folds for each of 1 candidates, totalling 5 fits
Fitting 5 folds for each of 1 candidates, totalling 5 fits
Fitting 5 folds for each of 1 candidates, totalling 5 fits
Fitting 5 folds for each of 1 candidates, totalling 5 fits
Fitting 5 folds for each of 1 candidates, totalling 5 fits
Fitting 5 folds for each of 1 candidates, totalling 5 fits
Fitting 5 folds for each of 1 candidates, totalling 5 fits
Fitting 5 folds for each of 1 candidates, totalling 5 fits
Fitting 5 folds for each of 1 candidates, totalling 5 fits
Fitting 5 folds for each of 1 candidates, totalling 5 fits
Fitting 5 folds for each of 1 candidates, totalling 5 fits
Fitting 5 folds for each of 1 candidates, totalling 5 fits
Fitting 5 folds for each of 1 candidates, totalling 5 fits
Fitting 5 folds for each of 1 candidates, totalling 5 fits
Fitting 5 folds for each of 1 candidates, totalling 5 fits
Fitting 5 folds for each of 1 candidates, totalling 5 fits
Fitting 5 folds for each of 1 candidates, totalling 5 fits
Fitting 5 folds for each of 1 candidates, totalling 5 fits
Fitting 5 folds for each of 1 candidates, totalling 5 fits
Fitting 5 folds for each of 1 candidates, totalling 5 fits
Fitting 5 folds for each of 1 candidates, totalling 5 fits
Fitting 5 folds for each of 1 candidates, totalling 5 fits
Fitting 5 folds for each of 1 candidates, totalling 5 fits
Fitting 5 folds for each of 1 candidates, totalling 5 fits
Fitting 5 folds for each of 1 candidates, totalling 5 fits
Fitting 5 folds for each of 1 candidates, totalling 5 fits
Fitting 5 folds for each of 1 candidates, totalling 5 fits
Fitting 5 folds for each of 1 candidates, totalling 5 fits
Fitting 5 folds for each of 1 candidates, totalling 5 fits
Fitting 5 folds for each of 1 candidates, totalling 5 fits
Fitting 5 folds for each of 1 candidates, totalling 5 fits
Fitting 5 folds for each of 1 candidates, totalling 5 fits
Fitting 5 folds for each of 1 candidates, totalling 5 fits
Fitting 5 folds for each of 1 candidates, totalling 5 fits
Fitting 5 folds for each of 1 candidates, totalling 5 fits
Fitting 5 folds for each of 1 candidates, totalling 5 fits
Fitting 5 folds for each of 1 candidates, totalling 5 fits
Fitting 5 folds for each of 1 candidates, totalling 5 fits
Fitting 5 folds for each of 1 candidates, totalling 5 fits
Fitting 5 folds for each of 1 candidates, totalling 5 fits
Fitting 5 folds for each of 1 candidates, totalling 5 fits
Fitting 5 folds for each of 1 candidates, totalling 5 fits
Fitting 5 folds for each of 1 candidates, totalling 5 fits
Fitting 5 folds for each of 1 candidates, totalling 5 fits
Fitting 5 folds for each of 1 candidates, totalling 5 fits
Fitting 5 folds for each of 1 candidates, totalling 5 fits
Fitting 5 folds for each of 1 candidates, totalling 5 fits
Fitting 5 folds for each of 1 candidates, totalling 5 fits
Fitting 5 folds for each of 1 candidates, totalling 5 fits
Fitting 5 folds for each of 1 candidates, totalling 5 fits
Fitting 5 folds for each of 1 candidates, totalling 5 fits
Fitting 5 folds for each of 1 candidates, totalling 5 fits
Fitting 5 folds for each of 1 candidates, totalling 5 fits
Fitting 5 folds for each of 1 candidates, totalling 5 fits
Fitting 5 folds for each of 1 candidates, totalling 5 fits
Fitting 5 folds for each of 1 candidates, totalling 5 fits
Fitting 5 folds for each of 1 candidates, totalling 5 fits
Fitting 5 folds for each of 1 candidates, totalling 5 fits
Completed in 638s

Best parameters:
OrderedDict({'xgb__colsample_bytree': 0.9, 'xgb__gamma': 2.866996421367216, 'xgb__learning_rate': 0.09325138680135805, 'xgb__max_depth': 2, 'xgb__min_child_weight': 10, 'xgb__n_estimators': 108, 'xgb__reg_alpha': 1.0, 'xgb__reg_lambda': 24.940415534287922, 'xgb__scale_pos_weight': 4.0, 'xgb__subsample': 0.9})
```

In [21]:

```
# ─────────────────────────────────────────────────────────────
# CV Metrics — Out-of-Fold (mean ± SD)
# ─────────────────────────────────────────────────────────────
y_pred_oof  = cross_val_predict(best_model, X_train_selected, y_train, cv=cv_stratified)
y_proba_oof = cross_val_predict(best_model, X_train_selected, y_train,
                                cv=cv_stratified, method='predict_proba')[:, 1]

# Optimal threshold (Youden index) on OOF predictions
fpr_cv, tpr_cv, thresholds_cv = roc_curve(y_train, y_proba_oof)
best_threshold_cv = thresholds_cv[np.argmax(tpr_cv - fpr_cv)]
print(f"Optimal threshold (Youden, OOF): {best_threshold_cv:.3f}")

cv_metrics = {'MCC': [], 'Balanced Accuracy': [], 'Sensitivity': [], 'Specificity': [], 'ROC-AUC': []}

for train_idx, val_idx in cv_stratified.split(X_train_selected, y_train):
    y_val       = y_train.iloc[val_idx]
    y_val_proba = y_proba_oof[val_idx]
    y_val_pred  = (y_val_proba >= best_threshold_cv).astype(int)
    tn, fp, fn, tp = confusion_matrix(y_val, y_val_pred).ravel()
    cv_metrics['Sensitivity'].append(tp / (tp + fn))
    cv_metrics['Specificity'].append(tn / (tn + fp))
    cv_metrics['Balanced Accuracy'].append(balanced_accuracy_score(y_val, y_val_pred))
    cv_metrics['MCC'].append(matthews_corrcoef(y_val, y_val_pred))
    cv_metrics['ROC-AUC'].append(roc_auc_score(y_val, y_val_proba))

cv_results_df = pd.DataFrame({
    'Metric': list(cv_metrics.keys()),
    'Mean'  : [np.mean(cv_metrics[m]) for m in cv_metrics],
    'SD'    : [np.std(cv_metrics[m], ddof=1) for m in cv_metrics],
    '95% CI Lower': [np.percentile(cv_metrics[m], 2.5) for m in cv_metrics],
    '95% CI Upper': [np.percentile(cv_metrics[m], 97.5) for m in cv_metrics],
})
cv_results_df.to_csv(os.path.join(TABLES_PATH, 'Table_XGB_CV_metrics.csv'), index=False)
print("\nCV metrics (mean ± SD):")
display(cv_results_df)
```

```
Optimal threshold (Youden, OOF): 0.500

CV metrics (mean ± SD):
```

|  | Metric | Mean | SD | 95% CI Lower | 95% CI Upper |
| --- | --- | --- | --- | --- | --- |
| 0 | MCC | 0.306411 | 0.069001 | 0.214934 | 0.371045 |
| 1 | Balanced Accuracy | 0.624526 | 0.027003 | 0.585596 | 0.651923 |
| 2 | Sensitivity | 0.961667 | 0.056396 | 0.880833 | 1.000000 |
| 3 | Specificity | 0.287385 | 0.048862 | 0.234615 | 0.354769 |
| 4 | ROC-AUC | 0.598128 | 0.069912 | 0.513423 | 0.677179 |

In [22]:

```
# ─────────────────────────────────────────────────────────────
# Final Evaluation — Test Set
# Threshold: Youden index computed on OOF predictions (train set)
# → avoids any leakage from the test set
# ─────────────────────────────────────────────────────────────
y_proba_test = best_model.predict_proba(X_test_selected)[:, 1]

# Apply OOF-derived threshold (no leakage)
y_pred_test = (y_proba_test >= best_threshold_cv).astype(int)

# Variables for bootstrap (next section)
y_pred_final  = y_pred_test
y_proba_final = y_proba_test

tn, fp, fn, tp = confusion_matrix(y_test, y_pred_test).ravel()
sensitivity_test = tp / (tp + fn)
specificity_test = tn / (tn + fp)

# ── Youden threshold report ───────────────────────────────────
print('=' * 55)
print(f'Optimal threshold (Youden index, OOF): {best_threshold_cv:.3f}')
print(f'  Sensitivity at threshold : {sensitivity_test:.3f}')
print(f'  Specificity at threshold : {specificity_test:.3f}')
print('=' * 55)

test_metrics_df = pd.DataFrame({
    'Metric': [
        'ROC-AUC', 'AUC-PR', 'MCC', 'Balanced Accuracy',
        'Sensitivity', 'Specificity', 'Brier Score',
        'Decision Threshold (Youden)'
    ],
    'Value': [
        roc_auc_score(y_test, y_proba_test),
        average_precision_score(y_test, y_proba_test),
        matthews_corrcoef(y_test, y_pred_test),
        balanced_accuracy_score(y_test, y_pred_test),
        sensitivity_test,
        specificity_test,
        brier_score_loss(y_test, y_proba_test),
        best_threshold_cv,
    ]
})
test_metrics_df.to_csv(os.path.join(TABLES_PATH, 'Table_XGB_test_metrics.csv'), index=False)
print('\nTest set metrics:')
display(test_metrics_df)
```

```
=======================================================
Optimal threshold (Youden index, OOF): 0.500
  Sensitivity at threshold : 0.737
  Specificity at threshold : 0.545
=======================================================

Test set metrics:
```

|  | Metric | Value |
| --- | --- | --- |
| 0 | ROC-AUC | 0.671451 |
| 1 | AUC-PR | 0.501644 |
| 2 | MCC | 0.273701 |
| 3 | Balanced Accuracy | 0.641148 |
| 4 | Sensitivity | 0.736842 |
| 5 | Specificity | 0.545455 |
| 6 | Brier Score | 0.259471 |
| 7 | Decision Threshold (Youden) | 0.500471 |

In [23]:

```
# ─────────────────────────────────────────────────────────────
# Calibration Curve + Brier Score
# ─────────────────────────────────────────────────────────────
prob_true, prob_pred = calibration_curve(y_test, y_proba_test, n_bins=10)

fig, ax = plt.subplots(figsize=(7, 6))
ax.plot(prob_pred, prob_true, marker='o', lw=2, label=f'XGBoost (Brier={brier_score_loss(y_test, y_proba_test):.3f})')
ax.plot([0, 1], [0, 1], linestyle='--', color='gray', label='Perfect Calibration')
ax.set_xlabel('Predicted Probability')
ax.set_ylabel('Observed Frequency')
ax.set_title('Calibration Curve — Optimized XGBoost')
ax.legend()
ax.grid(True, alpha=0.3)
plt.tight_layout()
plt.savefig(os.path.join(FIGURES_PATH, 'FigS2_calibration_curve.png'), **SAVEFIG_KW)
plt.show()
```

In [24]:

```
# ─────────────────────────────────────────────────────────────
# Bootstrap 95% Confidence Intervals (2000 resamples)
# Based on the optimised model (best_model)
# ─────────────────────────────────────────────────────────────
import numpy as np
from sklearn.metrics import confusion_matrix, balanced_accuracy_score, matthews_corrcoef, roc_auc_score

n_bootstrap = 2000
rng = np.random.RandomState(SEED)

mcc_scores, sens_scores, spec_scores, ba_scores, roc_scores = [], [], [], [], []

# Conversion pour éviter les problèmes d'index avec Pandas
y_test_arr = y_test.values if hasattr(y_test, 'iloc') else y_test

for i in range(n_bootstrap):
    # L'erreur de syntaxe était ici : le texte "Best parameters..." a été supprimé
    indices = rng.randint(0, len(y_test_arr), len(y_test_arr))

    y_true_b = y_test_arr[indices]
    if len(np.unique(y_true_b)) < 2:
        continue

    y_prob_b  = y_proba_final[indices]
    y_pred_b  = y_pred_final[indices]

    tn, fp, fn, tp = confusion_matrix(y_true_b, y_pred_b).ravel()

    sens_scores.append(tp / (tp + fn) if (tp + fn) > 0 else 0)
    spec_scores.append(tn / (tn + fp) if (tn + fp) > 0 else 0)
    ba_scores.append(balanced_accuracy_score(y_true_b, y_pred_b))
    mcc_scores.append(matthews_corrcoef(y_true_b, y_pred_b))
    roc_scores.append(roc_auc_score(y_true_b, y_prob_b))

def ci95(scores):
    return np.percentile(scores, 2.5), np.percentile(scores, 97.5)

print("Bootstrap 95% Confidence Intervals (2000 resamples — optimised model)")
print('-' * 55)
for name, scores in [
    ('ROC-AUC',           roc_scores),
    ('MCC',               mcc_scores),
    ('Balanced Accuracy', ba_scores),
    ('Sensitivity',       sens_scores),
    ('Specificity',       spec_scores),
]:
    lo, hi = ci95(scores)
    print(f"{name:<22} [{lo:.3f} — {hi:.3f}]")
```

```
Bootstrap 95% Confidence Intervals (2000 resamples — optimised model)
-------------------------------------------------------
ROC-AUC                [0.514 — 0.818]
MCC                    [0.018 — 0.517]
Balanced Accuracy      [0.510 — 0.770]
Sensitivity            [0.533 — 0.938]
Specificity            [0.375 — 0.710]
```

### 🔁 Nested CV — Validation Complémentaire¶

> This cell implements a lightweight nested cross-validation (outer 5-fold / inner BayesSearch 30 iterations)
> to verify the absence of optimisation bias in the main pipeline.

> If outer metrics ≈ main pipeline metrics → HPO is not over-fitting.

> *This cell is independent of the main pipeline and does not replace the final model.*

> ⚠️ **Estimated runtime: 15–30 min.**

In [25]:

```
# ─────────────────────────────────────────────────────────────
# Lightweight Nested CV
# Outer: 5-fold stratified | Inner: BayesSearch 30 iterations
# ─────────────────────────────────────────────────────────────
outer_cv = StratifiedKFold(n_splits=5, shuffle=True, random_state=SEED)
inner_cv = StratifiedKFold(n_splits=5, shuffle=True, random_state=SEED)

nested_metrics = {'MCC': [], 'ROC-AUC': [], 'Sensitivity': [], 'Specificity': []}

for fold_idx, (train_idx, val_idx) in enumerate(outer_cv.split(X_train_selected, y_train)):
    print(f"  Outer fold {fold_idx+1}/5...")

    X_tr, X_val = X_train_selected.iloc[train_idx], X_train_selected.iloc[val_idx]
    y_tr, y_val = y_train.iloc[train_idx],          y_train.iloc[val_idx]

    # Inner loop : optimisation
    inner_pipeline = ImbPipeline([
        ('xgb', xgb.XGBClassifier(random_state=SEED, eval_metric='logloss', n_jobs=-1))
    ])
    inner_search = BayesSearchCV(
        estimator=inner_pipeline,
        search_spaces=param_space_xgb,
        n_iter=30,
        cv=inner_cv,
        scoring=make_scorer(matthews_corrcoef),
        n_jobs=-1,
        random_state=SEED,
        verbose=0
    )
    inner_search.fit(X_tr, y_tr)
    best_fold_model = inner_search.best_estimator_

    # Outer fold evaluation
    y_val_proba = best_fold_model.predict_proba(X_val)[:, 1]
    fpr_, tpr_, thr_ = roc_curve(y_val, y_val_proba)
    thresh_ = thr_[np.argmax(tpr_ - fpr_)]
    y_val_pred = (y_val_proba >= 0.5).astype(int)
    tn, fp, fn, tp = confusion_matrix(y_val, y_val_pred).ravel()
    nested_metrics['MCC'].append(matthews_corrcoef(y_val, y_val_pred))
    nested_metrics['ROC-AUC'].append(roc_auc_score(y_val, y_val_proba))
    nested_metrics['Sensitivity'].append(tp / (tp + fn))
    nested_metrics['Specificity'].append(tn / (tn + fp))

nested_df = pd.DataFrame({
    'Metric': list(nested_metrics.keys()),
    'Mean'  : [np.mean(nested_metrics[m]) for m in nested_metrics],
    'SD'    : [np.std(nested_metrics[m], ddof=1) for m in nested_metrics],
})
nested_df.to_csv(os.path.join(TABLES_PATH, 'Table_nested_CV_metrics.csv'), index=False)
print("\nNested CV — Results (outer 5-fold):")
display(nested_df)
print("\n→ Comparer avec les métriques CV du pipeline principal (Section 6).")
print("  If values are close, HPO is not over-fitting.")
```

```
  Outer fold 1/5...
  Outer fold 2/5...
  Outer fold 3/5...
  Outer fold 4/5...
  Outer fold 5/5...

Nested CV — Results (outer 5-fold):
```

|  | Metric | Mean | SD |
| --- | --- | --- | --- |
| 0 | MCC | 0.111651 | 0.075780 |
| 1 | ROC-AUC | 0.614397 | 0.049718 |
| 2 | Sensitivity | 0.710833 | 0.152400 |
| 3 | Specificity | 0.395692 | 0.132760 |

```
→ Comparer avec les métriques CV du pipeline principal (Section 6).
  If values are close, HPO is not over-fitting.
```

---

## 📈 Section 7 — Interpretability & Publication Figures¶

> SHAP beeswarm, SHAP waterfall (highest-risk patient), SHAP bar (% contribution),
> ROC curves comparing 4 models.

In [26]:

```
# ─────────────────────────────────────────────────────────────
# SHAP — Optimised model (selected features)
# ─────────────────────────────────────────────────────────────
explainer   = shap.Explainer(xgb_model, X_train_selected)
shap_values = explainer(X_test_selected)

print(f"SHAP values shape: {shap_values.values.shape}")
print(f"Features         : {X_test_selected.shape[1]}")
```

```
SHAP values shape: (52, 36)
Features         : 36
```

In [27]:

```
# ─────────────────────────────────────────────────────────────
# Figure S1 — SHAP Beeswarm (impact + direction)
# ─────────────────────────────────────────────────────────────
#shap.summary_plot(shap_values, X_test_selected, show=False)
#plt.title('SHAP Summary — Optimized XGBoost', fontsize=12)
#plt.savefig(os.path.join(FIGURES_PATH, 'FigS1_SHAP_beeswarm.png'), **SAVEFIG_KW)
#plt.show()
# ─────────────────────────────────────────────────────────────
# Figure S1 — SHAP Beeswarm (impact + direction) & Pourcentages
# ─────────────────────────────────────────────────────────────
# 1. Génération et sauvegarde du graphique Beeswarm
shap.summary_plot(shap_values, X_test_selected, show=False)
plt.title('SHAP Summary — Optimized XGBoost', fontsize=12)
plt.savefig(os.path.join(FIGURES_PATH, 'FigS1_SHAP_beeswarm.png'), **SAVEFIG_KW)
plt.show()

# 2. Extraction des valeurs SHAP
if hasattr(shap_values, 'values'):
    vals = np.abs(shap_values.values)
else:
    vals = np.abs(shap_values)

# 3. Calcul de l'impact moyen absolu par caractéristique
mean_abs_shap = np.mean(vals, axis=0)

# 4. Calcul du pourcentage d'impact relatif
impact_percentages = (mean_abs_shap / np.sum(mean_abs_shap)) * 100

# 5. Création du DataFrame pour l'affichage
importance_df = pd.DataFrame({
    'Feature': X_test_selected.columns,
    'Mean_Absolute_Impact': mean_abs_shap,
    'Impact_Percentage (%)': impact_percentages
})

# Tri par ordre décroissant et arrondissement
importance_df = importance_df.sort_values(by='Impact_Percentage (%)', ascending=False).reset_index(drop=True)
importance_df['Impact_Percentage (%)'] = importance_df['Impact_Percentage (%)'].round(2)
importance_df['Mean_Absolute_Impact'] = importance_df['Mean_Absolute_Impact'].round(4)

# 6. Affichage
print("\n📊 --- Impact global des caractéristiques en pourcentage ---")
display(importance_df)
```

```
📊 --- Impact global des caractéristiques en pourcentage ---
```

|  | Feature | Mean\_Absolute\_Impact | Impact\_Percentage (%) |
| --- | --- | --- | --- |
| 0 | HbA1c | 0.4419 | 24.63 |
| 1 | Microalbuminuria | 0.2864 | 15.96 |
| 2 | Autonomic Neuropathy | 0.2703 | 15.06 |
| 3 | BMI | 0.2008 | 11.19 |
| 4 | DN4 Score | 0.0887 | 4.94 |
| 5 | Fasting Glucose | 0.0775 | 4.32 |
| 6 | Insulin | 0.0569 | 3.17 |
| 7 | MCHC | 0.0477 | 2.66 |
| 8 | WBC | 0.0443 | 2.47 |
| 9 | Diabetes Duration | 0.0429 | 2.39 |
| 10 | Systolic BP | 0.0424 | 2.36 |
| 11 | MCV | 0.0386 | 2.15 |
| 12 | eGFR | 0.0314 | 1.75 |
| 13 | Haemoglobin | 0.0234 | 1.30 |
| 14 | Metformin Duration | 0.0224 | 1.25 |
| 15 | MCH | 0.0192 | 1.07 |
| 16 | Erectile Dysfunction | 0.0140 | 0.78 |
| 17 | Platelets | 0.0125 | 0.70 |
| 18 | Diastolic BP | 0.0123 | 0.68 |
| 19 | ALT | 0.0116 | 0.64 |
| 20 | NIS Sensitivity Score | 0.0091 | 0.51 |
| 21 | Hypertension | 0.0000 | 0.00 |
| 22 | Age | 0.0000 | 0.00 |
| 23 | Sex | 0.0000 | 0.00 |
| 24 | Proton Pump Inhibitor | 0.0000 | 0.00 |
| 25 | Girerd Score | 0.0000 | 0.00 |
| 26 | Sulfonylureas | 0.0000 | 0.00 |
| 27 | Macroangiopathy | 0.0000 | 0.00 |
| 28 | Microangiopathy | 0.0000 | 0.00 |
| 29 | Peripheral Neuropathy | 0.0000 | 0.00 |
| 30 | Diabetic Nephropathy | 0.0000 | 0.00 |
| 31 | Metformin Daily Dose | 0.0000 | 0.00 |
| 32 | Metformin Cumulative Dose | 0.0000 | 0.00 |
| 33 | NIS Total Score | 0.0000 | 0.00 |
| 34 | NIS Reflexes Score | 0.0000 | 0.00 |
| 35 | AST | 0.0000 | 0.00 |

In [28]:

```
# ─────────────────────────────────────────────────────────────
# Figure S3 — SHAP Waterfall (highest-risk patient)
# ─────────────────────────────────────────────────────────────
highest_risk_idx = np.argmax(y_proba_test)

shap.waterfall_plot(shap_values[highest_risk_idx], show=False)
plt.title(
    f'SHAP Waterfall — Highest Risk Patient '
    f'(predicted prob = {y_proba_test[highest_risk_idx]:.2f})',
    fontsize=11
)
plt.savefig(os.path.join(FIGURES_PATH, 'FigS3_SHAP_waterfall.png'), **SAVEFIG_KW)
plt.show()
```

In [29]:

```
# ─────────────────────────────────────────────────────────────
# Figure 5 — SHAP Bar Chart (% contribution per feature)
# ─────────────────────────────────────────────────────────────
try:
    features = list(xgb_model.feature_names_in_)
except AttributeError:
    features = list(X_test_selected.columns)

mean_abs_shap = np.abs(shap_values.values).mean(axis=0)
feature_importance = pd.DataFrame({
    'Feature'     : features,
    'MeanAbsSHAP' : mean_abs_shap
}).sort_values('MeanAbsSHAP', ascending=False)

feature_importance['Percentage'] = (
    feature_importance['MeanAbsSHAP'] / feature_importance['MeanAbsSHAP'].sum() * 100
)
feature_importance.to_csv(os.path.join(TABLES_PATH, 'Table_SHAP_importance.csv'), index=False)

fig, ax = plt.subplots(figsize=(10, max(6, len(features) * 0.35)))
ax.barh(feature_importance['Feature'], feature_importance['Percentage'],
        color='#3498DB', edgecolor='white')
ax.set_xlabel('Percentage Contribution (%)')
ax.set_title('Feature Importance — Mean Absolute SHAP Values', fontsize=13, fontweight='bold')
ax.invert_yaxis()
ax.grid(True, axis='x', alpha=0.3)
plt.tight_layout()
plt.savefig(os.path.join(FIGURES_PATH, 'Fig5_SHAP_bar_importance.png'), **SAVEFIG_KW)
plt.show()
```

In [30]:

```
# ─────────────────────────────────────────────────────────────
# Figure S4 — ROC Curves comparing 4 models
# ─────────────────────────────────────────────────────────────
colors_roc = {
    'XGBoost'                  : '#E74C3C',
    'Random Forest'            : '#2ECC71',
    'SVM'                      : '#9B59B6',
    'Logistic Regression (L2)' : '#3498DB',
}

fig, ax = plt.subplots(figsize=(8, 7))

for name, model in models.items():
    y_score = cross_val_predict(
        model, X_train_selected, y_train,
        cv=cv5, method='predict_proba'
    )[:, 1]
    fpr_, tpr_, _ = roc_curve(y_train, y_score)
    auc_ = roc_auc_score(y_train, y_score)
    ax.plot(fpr_, tpr_, lw=2, color=colors_roc[name], label=f'{name} (AUC = {auc_:.3f})')

ax.plot([0, 1], [0, 1], '--', color='gray', alpha=0.5, label='Random (AUC = 0.500)')
ax.set_xlabel('1 - Specificity', fontsize=13)
ax.set_ylabel('Sensitivity', fontsize=13)
ax.set_title('ROC Curves — 5-Fold CV Comparison\n4 Models (SHAP-selected features)', fontsize=13, fontweight='bold')
ax.legend(loc='lower right', fontsize=11)
ax.grid(True, alpha=0.3)
plt.tight_layout()
plt.savefig(os.path.join(FIGURES_PATH, 'FigS4_ROC_comparison.png'), **SAVEFIG_KW)
plt.show()
```

---

## ✅ Summary of generated output files¶

### Figures (`results/figures/`)¶

| Fichier | Description |
| --- | --- |
| `Fig1_B12_deficiency_distribution.png` | Target variable distribution and proportion |
| `Fig2_resampling_comparison.png` | Resampling strategy comparison |
| `Fig3_SHAP_baseline_importance.png` | Baseline SHAP importance (feature selection) |
| `Fig4_model_comparison.png` | 4-model comparison |
| `Fig5_SHAP_bar_importance.png` | Feature % contribution (final model) |
| `FigS1_SHAP_beeswarm.png` | SHAP beeswarm (direction + magnitude) |
| `FigS2_calibration_curve.png` | Calibration curve + Brier Score |
| `FigS3_SHAP_waterfall.png` | SHAP waterfall — highest-risk patient |
| `FigS4_ROC_comparison.png` | ROC curves — 4 models |

### Tables (`results/tables/`)¶

| Fichier | Description |
| --- | --- |
| `Table1_comparative_stats.csv` | Table 1 (comparative statistics by B12 status) |
| `Table_resampling_comparison.csv` | Resampling strategy comparison |
| `Table_model_comparison.csv` | 4-model comparison |
| `Table_XGB_CV_metrics.csv` | CV metrics (mean ± SD, 95% CI) |
| `Table_XGB_test_metrics.csv` | Test set metrics |
| `Table_nested_CV_metrics.csv` | Nested CV (complementary validation) |
| `Table_SHAP_importance.csv` | SHAP feature importance |

In [31]:

```
# Clinical profile of misclassified cases
groups = {
    'TN': (y_pred_test == 0) & (y_test.values == 0),
    'TP': (y_pred_test == 1) & (y_test.values == 1),
    'FN': (y_pred_test == 0) & (y_test.values == 1),
    'FP': (y_pred_test == 1) & (y_test.values == 0),
}

features = ['HbA1c', 'Microalbuminuria', 'Autonomic Neuropathy','BMI','DN4 Score']

# Retrieve retained feature column names
cols = [f for f in features if f in X_test_selected.columns]

for grp, mask in groups.items():
    n = mask.sum()
    print(f"\n{grp} (n={n})")
    for f in cols:
        vals = X_test_selected.loc[mask, f]
        print(f"  {f}: mean={vals.mean():.2f}, sd={vals.std():.2f}")
```

```
TN (n=18)
  HbA1c: mean=10.01, sd=1.77
  Microalbuminuria: mean=10.29, sd=10.59
  Autonomic Neuropathy: mean=0.00, sd=0.00
  BMI: mean=31.49, sd=4.12
  DN4 Score: mean=2.56, sd=2.33

TP (n=14)
  HbA1c: mean=7.42, sd=1.15
  Microalbuminuria: mean=26.87, sd=27.78
  Autonomic Neuropathy: mean=0.36, sd=0.50
  BMI: mean=29.74, sd=4.53
  DN4 Score: mean=1.86, sd=2.28

FN (n=5)
  HbA1c: mean=10.08, sd=2.22
  Microalbuminuria: mean=33.58, sd=56.11
  Autonomic Neuropathy: mean=0.00, sd=0.00
  BMI: mean=35.07, sd=5.84
  DN4 Score: mean=2.20, sd=1.79

FP (n=15)
  HbA1c: mean=7.59, sd=0.86
  Microalbuminuria: mean=26.08, sd=26.19
  Autonomic Neuropathy: mean=0.20, sd=0.41
  BMI: mean=30.95, sd=3.92
  DN4 Score: mean=1.87, sd=2.03
```

In [32]:

```
import pandas as pd

groups_dict = {
    'TN': pd.Series((y_pred_test == 0) & (y_test.values == 0), index=y_test.index),
    'TP': pd.Series((y_pred_test == 1) & (y_test.values == 1), index=y_test.index),
    'FN': pd.Series((y_pred_test == 0) & (y_test.values == 1), index=y_test.index),
    'FP': pd.Series((y_pred_test == 1) & (y_test.values == 0), index=y_test.index),
}

# Vérification
for grp, mask in groups_dict.items():
    print(f"{grp}: n={mask.sum()}")
```

```
TN: n=18
TP: n=14
FN: n=5
FP: n=15
```

In [33]:

```
import matplotlib.pyplot as plt
import matplotlib.patches as mpatches
import numpy as np
import os

groups = ['TN', 'TP', 'FN', 'FP']
n_each = [18, 14, 5, 15]  # v11: TN=18, TP=14, FN=5, FP=15
colors = ['#2E86AB', '#44BBA4', '#E94F37', '#F18F01']

# Top-5 SHAP features (v11): col name → display label
features_plot = ['HbA1c', 'Microalbuminuria', 'Autonomic Neuropathy','BMI','DN4 Score']

fig, axes = plt.subplots(1, 5, figsize=(16, 5))
fig.patch.set_facecolor('white')
x = np.arange(len(groups))
bar_w = 0.55

for ax, feature in zip(axes, features_plot):
    means, sds = [], []
    for grp, mask in groups_dict.items():
        vals = X_test_selected.loc[mask, feature]
        means.append(vals.mean())
        sds.append(vals.std())

    means, sds = np.array(means), np.array(sds)
    bars = ax.bar(x, means, width=bar_w, color=colors,
                  edgecolor='white', linewidth=1.2, zorder=3)
    ax.errorbar(x, means, yerr=sds, fmt='none',
                ecolor='#333333', elinewidth=1.5, capsize=5, zorder=4)

    ax.set_xticks(x)
    ax.set_xticklabels(groups, fontsize=9, fontweight='bold')
    ax.set_title(feature, fontsize=9.5, fontweight='bold', pad=8)
    ax.spines['top'].set_visible(False)
    ax.spines['right'].set_visible(False)
    ax.yaxis.grid(True, alpha=0.3, zorder=0)
    ax.set_axisbelow(True)
    bars[2].set_edgecolor('#C73E1D')
    bars[2].set_linewidth(2.5)

    # Annotate n after ylim is set
    ymin, ymax = ax.get_ylim()
    for xi, n in enumerate(n_each):
        ax.text(xi, ymin - 0.03*(ymax-ymin), f'n={n}',
                ha='center', va='top', fontsize=7.5, color='#555555')
    ax.set_ylim(ymin - 0.12*(ymax-ymin), ymax)

legend_patches = [mpatches.Patch(color=c, label=f'{g} (n={n})')
                  for g, c, n in zip(groups, colors, n_each)]
fig.legend(handles=legend_patches, loc='lower center', ncol=4,
           fontsize=9, frameon=True, bbox_to_anchor=(0.5, -0.04))

plt.tight_layout(rect=[0, 0.04, 1, 1])

# ── Save figure ─────────────────────────────────────────────────
try:
    fig_png = os.path.join(FIGURES_PATH, 'Fig3_misclassification_profile.png')
    fig_pdf = os.path.join(FIGURES_PATH, 'Fig3_misclassification_profile.pdf')

    plt.savefig(fig_png, **SAVEFIG_KW, facecolor='white', edgecolor='none')
    plt.savefig(fig_pdf, **SAVEFIG_KW, facecolor='white', edgecolor='none')

    print(f"✅ Figure 3 saved successfully to :\n → {fig_png}\n → {fig_pdf}")
except NameError:
    print("⚠️ FIGURES_PATH or SAVEFIG_KW not defined — fallback local save.")
    plt.savefig('Fig3_misclassification_profile.png', dpi=300, bbox_inches='tight', facecolor='white')

plt.show()
```

```
✅ Figure 3 saved successfully to :
 → outputs/figures/Fig3_misclassification_profile.png
 → outputs/figures/Fig3_misclassification_profile.pdf
```

In [34]:

```
groups_dict = {
    'TN': pd.Series((y_pred_test == 0) & (y_test.values == 0), index=y_test.index),
    'TP': pd.Series((y_pred_test == 1) & (y_test.values == 1), index=y_test.index),
    'FN': pd.Series((y_pred_test == 0) & (y_test.values == 1), index=y_test.index),
    'FP': pd.Series((y_pred_test == 1) & (y_test.values == 0), index=y_test.index),
}
```

In [35]:

```
print("Pipeline steps:", list(best_model.named_steps.keys()))
import shap

xgb_clf = best_model.named_steps['xgb']

explainer = shap.TreeExplainer(xgb_clf)
shap_values_test = explainer.shap_values(X_test_selected)

print("SHAP values shape:", shap_values_test.shape)
print("X_test_selected columns:", list(X_test_selected.columns))
```

```
Pipeline steps: ['xgb']
SHAP values shape: (52, 36)
X_test_selected columns: ['Sex', 'Age', 'Hypertension', 'Proton Pump Inhibitor', 'Diabetes Duration', 'Metformin Duration', 'Metformin Daily Dose', 'Metformin Cumulative Dose', 'Girerd Score', 'Sulfonylureas', 'Insulin', 'Macroangiopathy', 'Diabetic Nephropathy', 'Erectile Dysfunction', 'Autonomic Neuropathy', 'Peripheral Neuropathy', 'Microangiopathy', 'BMI', 'Systolic BP', 'Diastolic BP', 'DN4 Score', 'NIS Reflexes Score', 'NIS Sensitivity Score', 'NIS Total Score', 'Haemoglobin', 'MCV', 'MCH', 'MCHC', 'WBC', 'Platelets', 'Fasting Glucose', 'HbA1c', 'eGFR', 'Microalbuminuria', 'AST', 'ALT']
```

In [36]:

```
import matplotlib.pyplot as plt
import numpy as np

shap_df = pd.DataFrame(shap_values_test, columns=X_test_selected.columns)

hba1c_shap = shap_df['HbA1c'].values
hba1c_val  = X_test_selected['HbA1c'].values
metf_val   = X_test_selected['Metformin Cumulative Dose'].values

fig, ax = plt.subplots(figsize=(7, 5))

sc = ax.scatter(hba1c_val, hba1c_shap,
                c=metf_val, cmap='RdYlBu_r',
                alpha=0.85, edgecolors='white', linewidths=0.4, s=70, zorder=3)

cbar = plt.colorbar(sc, ax=ax)
cbar.set_label('Metformin Cumulative Dose (g)', fontsize=9)

ax.axhline(0, color='gray', lw=1.0, ls='--', alpha=0.5)
ax.set_xlabel('HbA1c (%)', fontsize=10)
ax.set_ylabel('SHAP value for HbA1c', fontsize=10)
ax.spines['top'].set_visible(False)
ax.spines['right'].set_visible(False)
ax.grid(True, alpha=0.25)

plt.tight_layout()
plt.savefig('Fig4a_SHAP_HbA1c_metformin.png', dpi=300, bbox_inches='tight', facecolor='white')
plt.show()
print("Done")
```

```
Done
```

In [37]:

```
import matplotlib.pyplot as plt
import matplotlib.patches as mpatches
from matplotlib.patches import FancyBboxPatch
import os

# ── Constants ────────────────────────────────────────────────────
W   = 12.0   # figure width
H   = 15.2   # figure height
BH  = 0.30   # half-height of each box
GAP = 0.18   # vertical gap between box edge and arrow

C = {
    'data':  '#2B2B2B',
    'split': '#2E86AB',
    'train': '#2E86AB',
    'test':  '#888888',
    'pre':   '#7B3F9E',
    'imbal': '#E07B00',
    'cmp':   '#3B3B6B',
    'shap1': '#C73E1D',
    'hpt':   '#3B3B6B',
    'nested':'#555555',
    'eval':  '#44BBA4',
    'shap2': '#E94F37',
    'arrow': '#333333',
    'lock':  '#888888',
}

fig, ax = plt.subplots(figsize=(W, H))
ax.set_xlim(0, W)
ax.set_ylim(0, H)
ax.axis('off')
fig.patch.set_facecolor('white')

def draw_box(x, y, w, label, color, fs=9.5):
    rect = FancyBboxPatch(
        (x - w/2, y - BH), w, 2*BH,
        boxstyle="round,pad=0.03,rounding_size=0.12",
        facecolor=color, edgecolor='white', linewidth=1.8, zorder=4
    )
    ax.add_patch(rect)
    ax.text(x, y, label, ha='center', va='center',
            fontsize=fs, fontweight='bold', color='white', zorder=5)

def v_arrow(x, y_from, y_to, color='#333333', lw=1.6):
    ax.annotate('',
        xy=(x, y_to), xytext=(x, y_from),
        arrowprops=dict(
            arrowstyle='->', color=color,
            lw=lw, mutation_scale=12,
            shrinkA=0, shrinkB=0
        ), zorder=3
    )

def line(x1, y1, x2, y2, color='#333333', lw=1.6, ls='-'):
    ax.plot([x1, x2], [y1, y2], color=color, lw=lw, ls=ls, zorder=3)

# ── Layout ───────────────────────────────────────────────────────
XL = 3.2    # left column (train pipeline)
XR = 9.2    # right column (test set)
XM = 6.2    # center (merge)
WB = 5.6    # box width for pipeline
WT = 4.4    # box width for train/test
WF = 9.5    # full-width box

# Y positions
# Actual notebook order:
#   Split → Imputation → Encoding → Imbalance comparison (Table 6)
#   → Baseline XGBoost + SHAP selection (50→36)
#   → Model comparison (4 models, no resampling)
#   → Bayesian Opt + scale_pos_weight → Nested CV → Final Eval → SHAP
YS = [
    14.60,  # 0  Full Dataset
    13.50,  # 1  Stratified Split
    12.40,  # 2  Train / Test
    11.30,  # 3  Missing Data Imputation
    10.20,  # 4  Categorical Encoding
     9.10,  # 5  Imbalance Strategy Comparison
     8.00,  # 6  SHAP Feature Selection
     6.90,  # 7  Model Comparison
     5.80,  # 8  Bayesian Opt + scale_pos_weight
     4.70,  # 9  Nested CV
     3.30,  # 10 Final Evaluation  (full-width, needs merge)
     1.00,  # 11 SHAP Interpretation
]

# 0 ── Full Dataset
draw_box(XM, YS[0], WF, "Full Dataset  (n=257 patients, 50 variables)", C['data'], fs=10.5)
v_arrow(XM, YS[0]-BH-GAP, YS[1]+BH+GAP)

# 1 ── Stratified Split
draw_box(XM, YS[1], WF, "Stratified 80/20 Split  (random_state=42)", C['split'], fs=10.5)

# Branch to Train / Test
y_branch = YS[1] - BH - GAP
y_hline  = y_branch - 0.22
line(XM, y_branch, XM, y_hline)
line(XL, y_hline,  XR, y_hline)
line(XL, y_hline,  XL, YS[2]+BH+GAP, C['split'])
line(XR, y_hline,  XR, YS[2]+BH+GAP, C['lock'])
ax.annotate('', xy=(XL, YS[2]+BH+GAP), xytext=(XL, YS[2]+BH+GAP+0.01),
            arrowprops=dict(arrowstyle='->', color=C['split'], lw=1.6,
                            mutation_scale=12, shrinkA=0, shrinkB=0), zorder=3)
ax.annotate('', xy=(XR, YS[2]+BH+GAP), xytext=(XR, YS[2]+BH+GAP+0.01),
            arrowprops=dict(arrowstyle='->', color=C['lock'], lw=1.6,
                            mutation_scale=12, shrinkA=0, shrinkB=0), zorder=3)

# 2 ── Train / Test boxes
draw_box(XL, YS[2], WT, "X_train  (n=205)", C['train'], fs=10)
draw_box(XR, YS[2], WT, "X_test  (n=52)  [LOCKED]", C['test'], fs=10)

# Locked dashed line for test set
y_lock_bot = 3.70
line(XR, YS[2]-BH-GAP, XR, y_lock_bot, C['lock'], lw=1.4, ls=(0, (4, 3)))
ax.text(XR, (YS[2]-BH-GAP + y_lock_bot)/2, "LOCKED",
        ha='center', va='center', fontsize=7.5, color=C['lock'], style='italic',
        bbox=dict(facecolor='white', edgecolor=C['lock'], boxstyle='round,pad=0.2',
                  linewidth=0.8, alpha=0.95), zorder=6)

# Arrow from train box down
v_arrow(XL, YS[2]-BH-GAP, YS[3]+BH+GAP, C['split'])

# 3 ── Missing Data Imputation
draw_box(XL, YS[3], WB,
         "Missing Data Imputation  (type-aware: mode / median / mean)",
         C['pre'], fs=9.0)
v_arrow(XL, YS[3]-BH-GAP, YS[4]+BH+GAP)

# 4 ── Categorical Encoding
draw_box(XL, YS[4], WB, "Categorical Encoding  (binary 0/1)", C['pre'], fs=9.5)
v_arrow(XL, YS[4]-BH-GAP, YS[5]+BH+GAP)

# 5 ── Imbalance Strategy Comparison  (exploratory, on all features)
draw_box(XL, YS[5], WB,
         "Imbalance Strategy Comparison  (Table 6)",
         C['imbal'], fs=9.0)
# Small annotation: what was compared
ax.text(XL, YS[5] - BH - 0.04,
        "BorderlineSMOTE  ·  ADASYN  ·  scale_pos_weight  →  scale_pos_weight selected",
        ha='center', va='top', fontsize=7.2, color='#666666', style='italic', zorder=5)
v_arrow(XL, YS[5]-BH-GAP-0.12, YS[6]+BH+GAP)

# 6 ── SHAP Feature Selection  (baseline model, fitted on X_train)
draw_box(XL, YS[6], WB,
         "SHAP Feature Selection  (50 → 36 features, baseline XGBoost)",
         C['shap1'], fs=9.0)
v_arrow(XL, YS[6]-BH-GAP, YS[7]+BH+GAP)

# 7 ── Model Comparison  (4 models, 5-fold CV, no resampling)
draw_box(XL, YS[7], WB,
         "Model Comparison  (4 algorithms, 5-fold CV)",
         C['cmp'], fs=9.5)
v_arrow(XL, YS[7]-BH-GAP, YS[8]+BH+GAP)

# 8 ── Bayesian Optimisation + scale_pos_weight
draw_box(XL, YS[8], WB,
         "Bayesian Opt.  (100 iter, MCC scoring) + scale_pos_weight=4.0",
         C['imbal'], fs=8.8)
v_arrow(XL, YS[8]-BH-GAP, YS[9]+BH+GAP)

# 9 ── Nested CV
draw_box(XL, YS[9], WB,
         "Nested Cross-Validation  (overfitting check)",
         C['nested'], fs=9.5)

# ── Merge ────────────────────────────────────────────────────────
y_merge = YS[9] - BH - GAP - 0.20
line(XL, YS[9]-BH-GAP, XL, y_merge, C['arrow'])
line(XR, y_lock_bot, XR, y_merge, C['lock'])
line(XL, y_merge, XR, y_merge, C['arrow'])
v_arrow(XM, y_merge, YS[10]+BH+GAP)

# 10 ── Final Evaluation
draw_box(XM, YS[10], WF,
         "Final Evaluation on Independent Test Set  (n=52)",
         C['eval'], fs=10.5)

# 11 ── SHAP Interpretation
v_arrow(XM, YS[10]-BH-GAP, YS[11]+BH+GAP)
draw_box(XM, YS[11], WF, "SHAP Interpretation — Final Model", C['shap2'], fs=10.5)

# ── Save ─────────────────────────────────────────────────────────
try:
    fig_png = os.path.join(FIGURES_PATH, 'Figure1_pipeline.png')
    fig_pdf = os.path.join(FIGURES_PATH, 'Figure1_pipeline.pdf')
    plt.savefig(fig_png, **SAVEFIG_KW, facecolor='white', edgecolor='none')
    plt.savefig(fig_pdf, **SAVEFIG_KW, facecolor='white', edgecolor='none')
    print(f"✅ Figure 1 saved:\n → {fig_png}\n → {fig_pdf}")
except NameError:
    print("⚠️ FIGURES_PATH or SAVEFIG_KW not defined — figure generated but not saved.")

plt.show()
```

```
✅ Figure 1 saved:
 → outputs/figures/Figure1_pipeline.png
 → outputs/figures/Figure1_pipeline.pdf
```
