# Supplementary figures and images for: Interpretable Machine Learning to Predict Metformin-Induced Vitamin B12 Deficiency: Association with Glycemic Control and Neuropathic Symptoms
Source: Metabolites. 2026 Mar 30;16(4):227. doi: 10.3390/metabo16040227 (PMC13118126; doi:10.3390/metabo16040227)

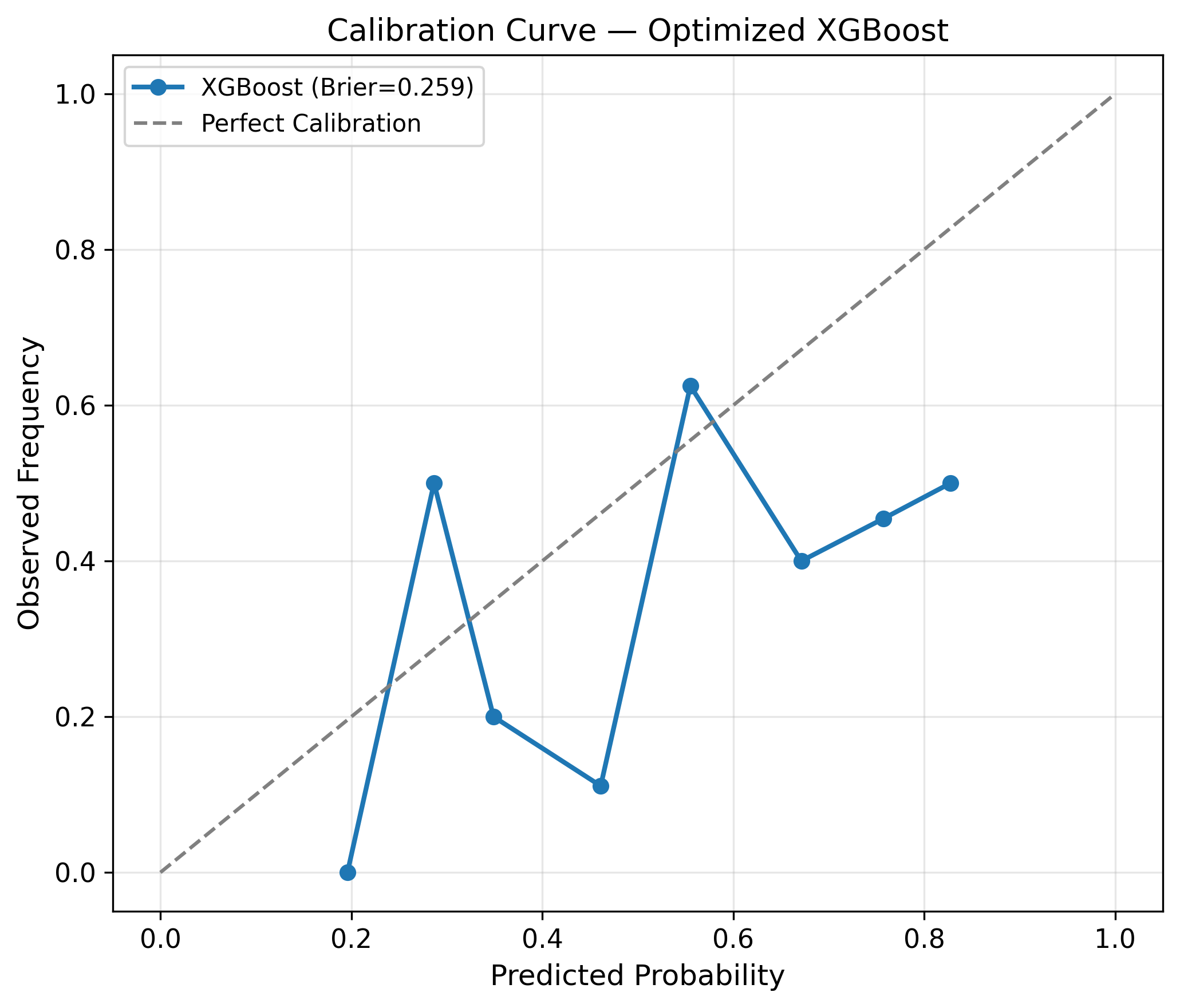

Supplement: Supplementary file 1 [file metabolites-16-00227-s001.zip › Supplemental Material/FigS2_calibration_curve.png]

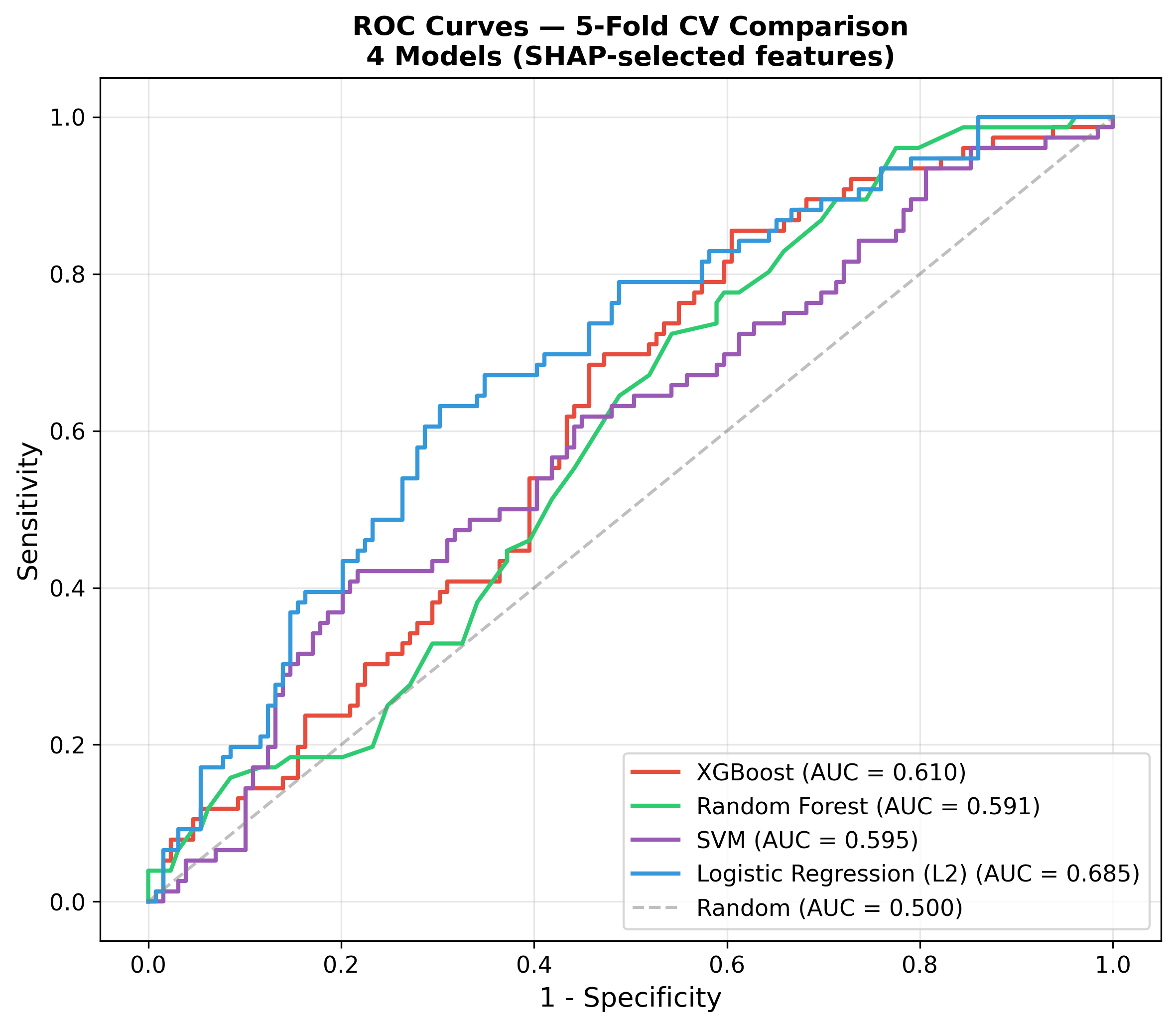

Supplement: Supplementary file 1 [file metabolites-16-00227-s001.zip › Supplemental Material/FigS4_ROC_comparison.png]
